# Biodegradable Nickel Disulfide Nanozymes with GSH-Depleting Function for High-Efficiency Photothermal-Catalytic Antibacterial Therapy

**DOI:** 10.1016/j.isci.2020.101281

**Published:** 2020-06-16

**Authors:** Xianwen Wang, Linxin Fan, Liang Cheng, Yanbin Sun, Xiyu Wang, Xiaoyan Zhong, Qianqian Shi, Fei Gong, Yu Yang, Yan Ma, Zhaohua Miao, Zhengbao Zha

**Affiliations:** 1 School of Food and Biological Engineering, Hefei University of Technology, Hefei 230009, P. R. China; 2 Institute of Functional Nano & Soft Materials (FUNSOM), Jiangsu Key Laboratory for Carbon-based Functional Materials and Devices, Soochow University, Suzhou 215123, P. R. China; 3 National Engineering Research Centre for Nanomedicine, College of Life Science and Technology, Huazhong University of Science and Technology, Wuhan 430074, P. R. China

**Keywords:** Medicine, Catalysis, Nanomaterials

## Abstract

Bacterial infections caused by pathogens have always been a thorny issue that threatens human health, and there is urgent need to develop a new generation of antimicrobial nano-agents and treatments. Herein, biodegradable nickel disulfide (ND) nanozymes as excellent antibacterial agents that integrate excellent photothermal performance, nano-catalysis property, and glutathione (GSH)-depleting function have been successfully constructed. The ND nanozymes can effectively catalyze the decomposition of H_2_O_2_ to produce ⋅OH, and the hyperthermia of ND nanozymes generated by photothermal therapy (PTT) can further increase its catalytic activity, which provides rapid and effective bacterial killing effect compared with nano-catalytic treatment or PTT alone. Surprisingly, the ND nanozymes have the ability of GSH consumption, thus enhancing its sterilization effect. Moreover, the ND nanozymes are biodegradable nanomaterials that do not cause any significant toxicity *in vivo*. Collectively, the ND nanozymes with excellent photothermal performance, catalytic activity, and GSH-depleting function are used for high-efficiency sterilization.

## Highlights

• ND nanozymes have good photothermal and catalysis effect and GSH-depleting function
• The multifunctional ND nanozymes have achieved satisfactory antibacterial effects
• The biodegradable ND nanozymes have a wide application in precise sterilization

## Graphical Abstract

**Figure:**
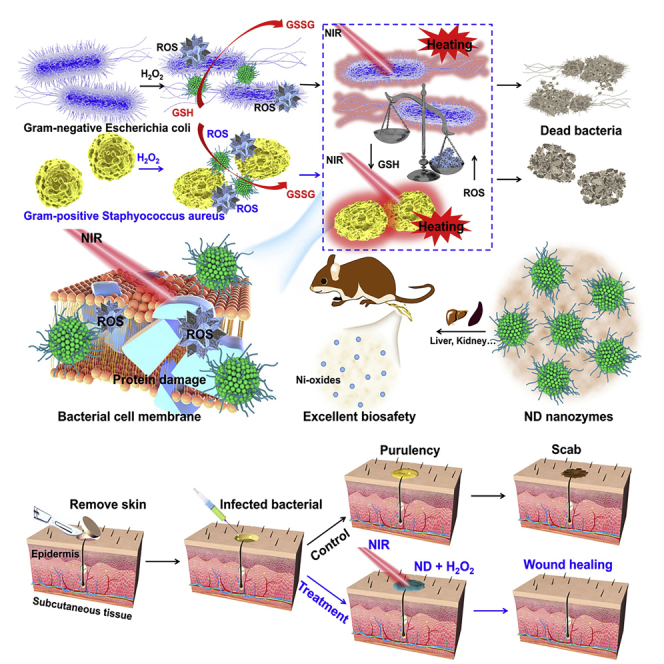

## Introduction

Bacterial infection has become a fatal worldwide health problem faced by human beings due to its high morbidity and mortality (Huo et al., 2019; Miao et al., 2019; Wang et al., 2019a; Wentao et al., 2019; Zhang et al., 2019b). The traditional methods to fight against bacterial infection are mainly based on antibiotics, metal ions, and quaternary ammonium ions, which are costly, toxic, and environmentally unfriendly, limiting their further use and conversion (Gao et al., 2018; Liu et al., 2020; Zhang et al., 2019a). Moreover, the excessive use of antibiotics leads to the generation of bacterial resistance, the treatment effect is inevitably reduced and even leads to the production of super-bacteria, thus causing a major threat to people's health and safety (Huo et al., 2019). Thus, it is of great significance to explore a new therapeutic strategy to more effectively and safely fight bacterial infections without having obvious side effects and resulting drug resistance (Liu et al., 2019b; Wang et al., 2019a).

Benefiting from the rapid rise and development of nanozymes, more attention has been paid to the study of using inorganic Fenton/Fenton-like reagents with horseradish peroxidase (HRP)-mimic ability to kill bacteria (Cao et al., 2019; Sang et al., 2019; Xi et al., 2019a). These reagents have been generally considered explored as excellent reactive HRP-like nanozymes, which can effectively catalyze the typical peroxidase-like reaction in the presence of H_2_O_2_, producing lots of reactive oxygen species (ROS) that have prominent oxidation effect (e.g., ⋅OH) (Huo et al., 2019; Wang et al., 2020a). These HRP-like nanozymes can be delivered to and accumulated at the pathological site through the drug delivery nanoplatforms, generating a large amount of ROS, which eventually leads to the dominant inhibition and destruction of drug-resistant bacterial cells (Huo et al., 2019). Unfortunately, HRP-like nanozymes limit their further application in antimicrobials for the following several reasons. (1) Most of the reported HRP-like nanozymes are non-degradable and cannot be removed from the body after sterilization, thus inevitably causing long-term toxicity *in vivo* (Zhang et al., 2019a). (2) Glutathione (GSH) is a tripeptide molecule that widely existed in bacteria, which played an important role in the antioxidant defense system of bacteria and effectively prevented oxidative stress from damaging cell components. Thus, GSH existing in bacteria can significantly weaken the bactericidal effect of peroxide-like nanomaterials (Yan et al., 2013). (3) As previously reported, it is difficult to completely eliminate bacteria by using HRP-like nanozymes alone (Liu et al., 2019b; Qing et al., 2019). The combination therapy based on sterilization is an effective strategy to improve the antibacterial efficiency owing to their effective synergistic effect (Liu et al., 2019c; Wu et al., 2019; Yang et al., 2019c). Hence, it is of great significance to develop biodegradable HRP-like nanozymes with multiple antibacterial function.

From the basic principle that increasing temperature can accelerate the chemical reaction rate, it is an effective strategy to improve the catalytic rate of HRP-like nanozymes by raising the temperature of bacterial infection site (Tang et al., 2017; Wang et al., 2019b, 2020b; Yang et al., 2019a). Among all the strategies currently reported for hyperthermia, photothermal therapy (PTT) is a new type of bactericidal therapy that employs photothermal agents (PTAs) to convert near-infrared light into overheating to destroy different types of pathogens and microorganisms, which is considered as one of the most promising treatment methods due to its less-invasive nature and high selectivity (Liu et al., 2019a). Therefore, developing novel HRP-like nanozymes with excellent photothermal properties will be a feasible strategy to improve the killing efficiency against bacteria (Xi et al., 2019b). To date, different HRP-like nanozymes including MoS_2_ (Yin et al., 2016) nanomaterials, CuFe_2_O_4_ (Liu et al., 2019b) nanoparticles, single-iron-atom nanocatalysts (Huo et al., 2019; Xu et al., 2019), Fe_3_O_4_ nanomaterials (Xu et al., 2018), carbon nanoparticles (Xi et al., 2019b), etc., have been reported for photothermal-catalytic antimicrobial therapy. However, the non-biodegradability of HRP-like nanozymes would lead to long-term toxicity *in vivo* (Yang et al., 2019b). More importantly, all these nanozymes have no GSH depletion function, which weakens their antibacterial effect. Based on this, the development of biodegradable HRP-like nanozymes with GSH-depleting function for photothermal-catalytic antimicrobial therapy is challenging and meaningful.

**Scheme 1:**
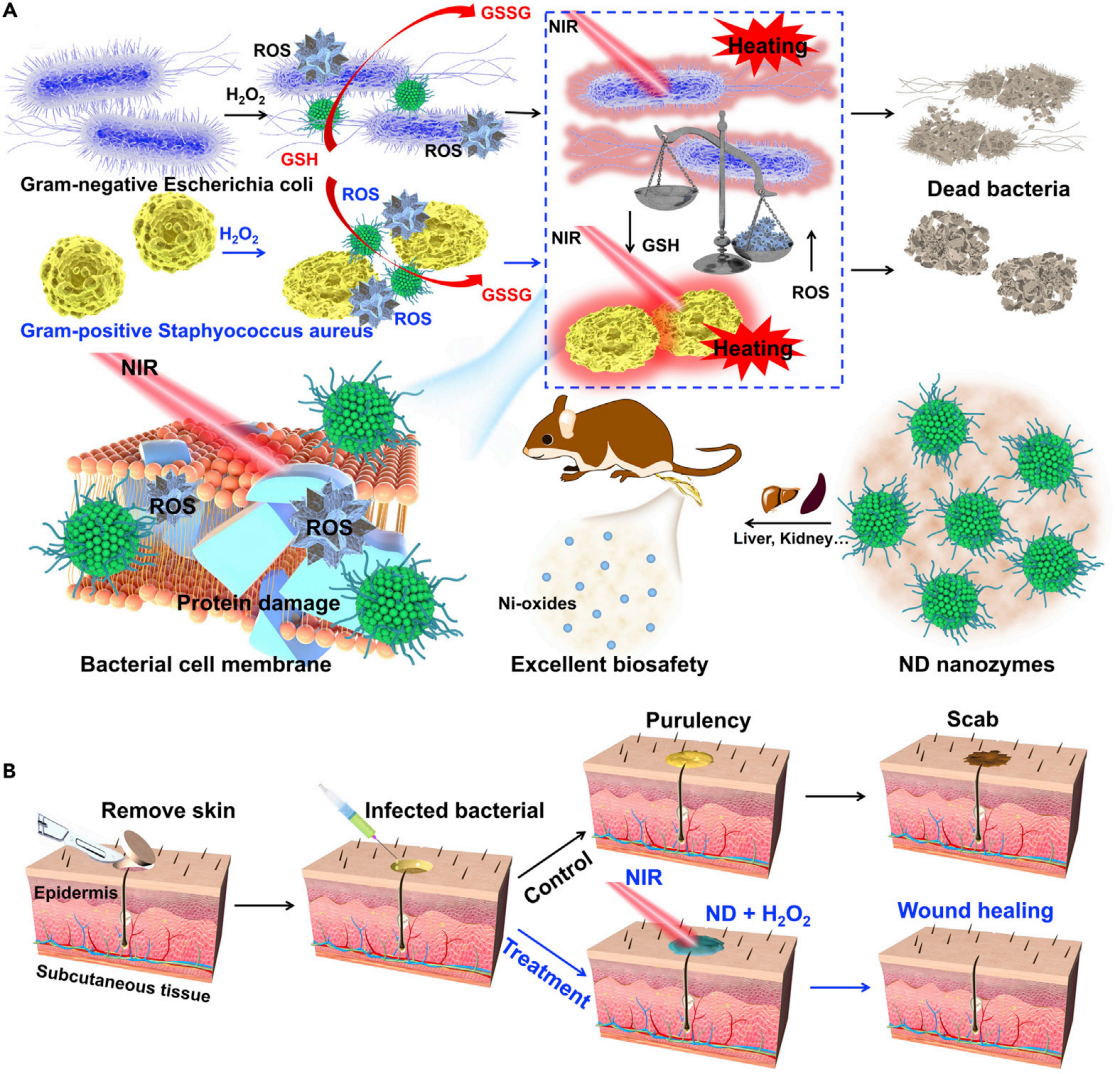
Scheme of the Biodegradable ND Nanozymes with GSH-depleting Function for High-efficiency Photothermal-catalytic Antibacterial Therapy (A) *in vitro* and (B) *in vivo*.

Nickel-based nanomaterials have been widely used in the field of nanomedicine owing to their good biocompatibility, prominent photothermal performance, and biodegradability (Liu et al., 2019d; Wang et al., 2017). It is worth noting that nickel-based nanomaterials are an excellent HRP-like nanozyme that can detect H_2_O_2_ and glucose by colorimetry (Chen et al., 2018; He et al., 2019). However, their peroxides-like catalytic activity and photothermal properties have not been reported for sterilization. Inspired by the outstanding peroxide activity and the photothermal properties of nickel-based nanomaterials, it is speculated that the combination of HRP-like catalytic activity and PTT may make up for the deficiency of single-mode antibacterial strategy and improve the antibacterial performance of the wound. Herein, monodisperse nickel disulfide (ND) nanozymes were fabricated through a facile solvothermal process, the as-prepared ND nanozymes had satisfactory near-infrared (NIR) absorption, high photothermal conversion efficiency, and excellent peroxide-like catalytic activity (Scheme 1A). The obtained ND nanozymes could generate ⋅OH through Fenton-like reaction in the presence of H_2_O_2_, and the catalytic activity of ND nanozymes was further enhanced by the mild photothermal performance, which has led to the achievement of photothermally enhanced catalytic bacterial treatment *in vitro*. Surprisingly, the ND nanoparticles could also act as another kind of enzyme (glutathione peroxidase mimetic, GSH-P_x_) that accelerated the consumption of GSH by oxidizing molecules and further weakened the bacteria through the GSH removal ability of ROS, thus enhancing its sterilization effect. More importantly, the experimental results of wound healing showed that the synergetic antibacterial nanoplatform could be easily used for wound disinfection (Scheme 1B). Most importantly, the ND nanozymes could quickly be removed from the body through urine and feces, due to the property of rapid biodegradability, without causing any significant toxicity through the systematic evaluation. Overall, the biodegradable ND nanozymes as multifunctional antibacterial agents have a broad prospect for accurate antibacterial application.

## Results

### Characterization of ND Nanozymes

**Figure 1:**
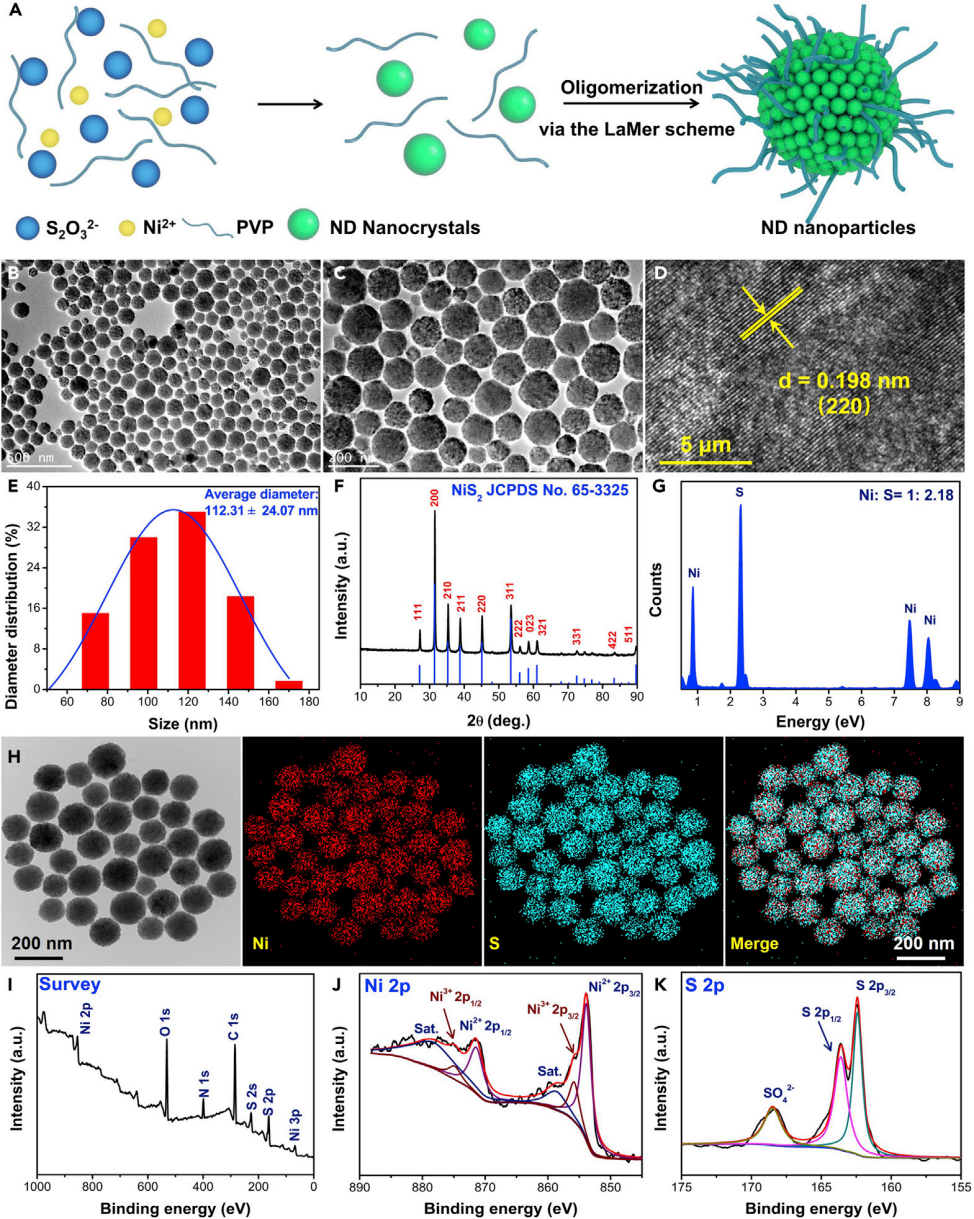
Characterization of the As-prepared ND Nanozymes (A–H) (A) Preparation scheme of ND nanoparticles, (B and C) TEM images, (D) high-resolution TEM image of ND nanozymes, (E) the size distribution determined from (B), (F) X-ray diffraction pattern, (G) energy-dispersive X-ray spectrum, and (H) scanning transmission electron microscopic elemental mapping (Ni, S, merge) of the ND nanozymes. (I–K) XPS characterization of ND nanozymes. The survey spectra (I) and the core level spectra of (J) Ni 2p and (K) S 2p. See also Figure S1.

Monodisperse and uniform ND nanozymes were successfully prepared by a facile PVP-assisted solvothermal method (Figure 1A), and the formation mechanism could be explained by the La Mer scheme based on the previous report (Wang et al., 2020b). Transmission electron microscopic (TEM) images of as-prepared ND nanozymes clearly revealed a uniform spherical morphology with an average diameter of 112.31 ± 24.07 nm (Figures 1B, 1C, and 1E). In addition, the TEM image further demonstrated that the ND nanozymes had highly ordered pore structures, which was due to the spherical accumulation of NiS_2_ nanocrystals (Figure 1C). High-resolution TEM image showed that the lattice spacing was measured to be ∼0.198 nm, corresponding to the (220) interplanar spacing of the cubic ND (Figure 1D). The diffraction rings of selected area electron diffraction (SAED) image indicated that the synthesized ND nanozymes had polycrystalline structure (Figure S1). The crystal structure and purity of the samples were also determined by X-ray diffraction. All the diffraction peaks corresponded to the cubic ND crystals (JCPDS No. 65-3325), suggesting the good crystallinity and purity of the synthesized ND nanozymes (Figure 1F). The mole ratio of Ni and S elements in ND was close to 1: 2, proved by energy-dispersive X-ray spectroscopic spectrum and the inductively coupled plasma optical emission spectroscopy (Figure 1F). In addition, field emission scanning transmission electron microscopic elemental mapping proved that the ND nanozymes were uniformly composed of Ni and S elements (Figure 1G). Moreover, X-ray photoelectron spectroscopy (XPS) was used to further confirm the chemical composition and the surface elemental states of ND nanozymes (Figures 1H–1J). The Ni, S, and O, C, and N elements existed in the full-scan XPS survey spectrum of ND nanozymes, where C, N, and O elements mainly come from the substrate (Figure 1I). In the Ni 2p spectra (Figure 1J), the peaks located at 871.49 and 853.85 eV correspond to Ni 2p_1/2_ and Ni 2p_3/2_ of the Ni^2+^ ions, respectively, along with two satellite peaks at about 874.90 and 859.22 eV. In the S 2p spectra (Figure 1K), the binding energies of S 2p_1/2_ and S 2p_3/2_ were clearly observed at about 162.44 and 163.67 eV of S elements, showing the presence of S-S bonds in the ND nanozymes (He et al., 2019). Besides, the peaks located at 168.41 eV were attributed to S-O bonds, which were caused by the surface oxidation of ND nanozymes. In addition, the zeta potential of the ND nanozymes was measured to be −9.80 ± 0.12 mV. All the aforementioned results demonstrated that ND nanozymes with high purity and quality were successfully prepared through a simple solvothermal method.

### Photothermal Performance of ND Nanozymes

**Figure 2:**
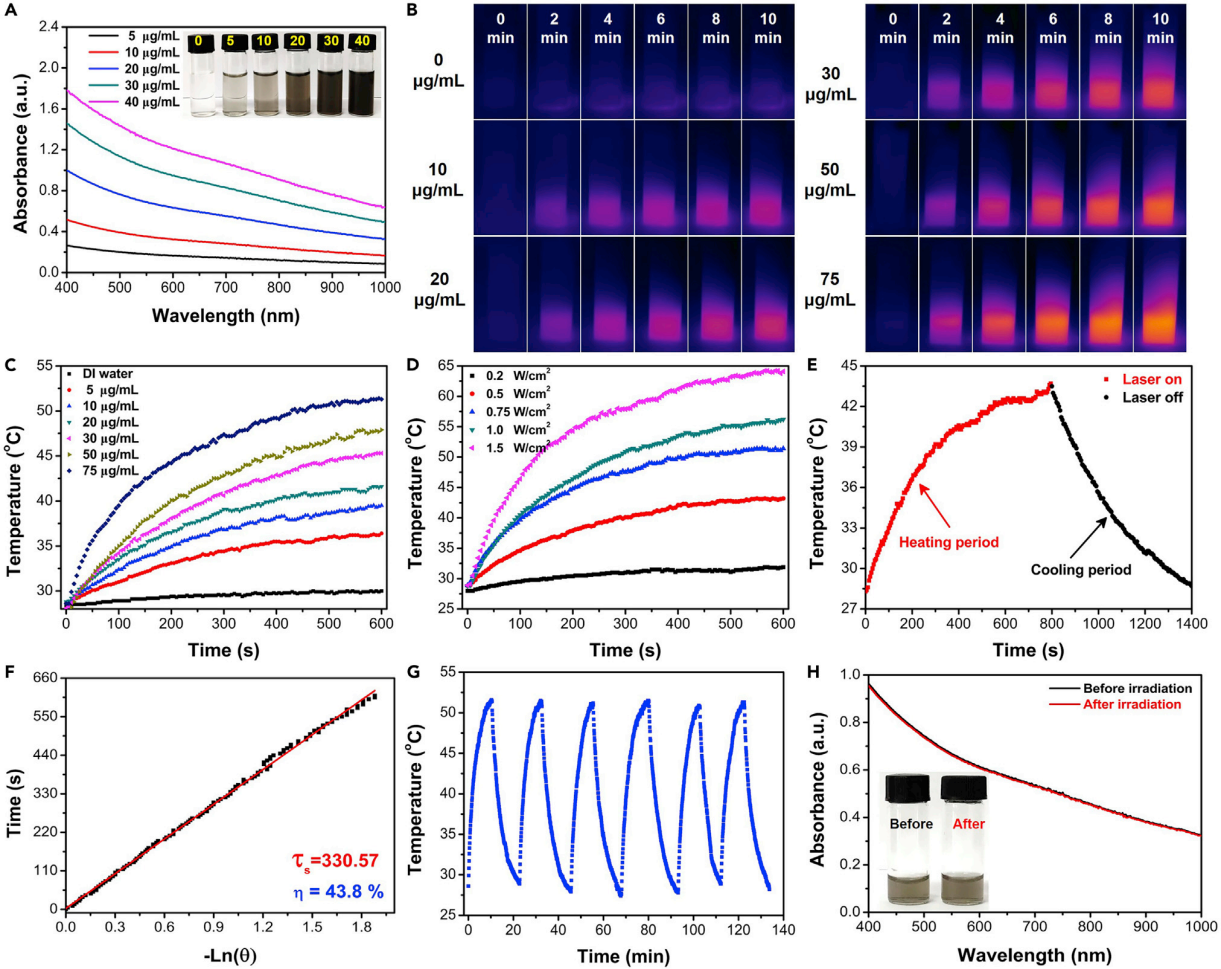
The Photothermal Effect of the As-prepared ND Nanozymes (A–C) (A) UV-vis-NIR spectra (insets: photographs of NPs with different concentrations), (B) NIR thermal images, and (C) the corresponding temperature change curves of ND nanozymes with gradient concentrations under NIR irradiation (808 nm, 0.75 W/cm^2^, 10 min). (D) Temperature heating curves under NIR irradiation with various power densities (ND nanozymes: 75 μg/mL, 808 nm, 10 min). (E) The temperature profile of ND nanozymes (20 μg/mL) irradiated by NIR laser (808 nm, 0.75 W/cm^2^) for 12 min, followed by natural cooling to room temperature. (F) The time constant was determined to be τ_s_ = 330.57 s, and photothermal conversion efficiency of ND nanozymes was as high as ∼43.8%. (G and H) (G) The photothermal stability (six laser on/off cycles) and (H) UV-vis-NIR spectra and photographs of the ND nanozymes before and after six laser on/off cycles. See also Figures S2–S7 and Table S1.

Good NIR absorbance and excellent photothermal conversion efficiency are the two most basic prerequisites of PTAs (Wang et al., 2017). Excitingly, the synthesized ND nanozymes exhibited wide NIR absorbance, which increased linearly with the enhancing concentration of ND nanozymes (Figure 2A). In addition, the mass extinction coefficient of ND nanozymes was ∼22.46 L g^−1^ cm^−1^ (Figure S2), much higher than those of most reported PTAs (Zhang et al., 2016), suggesting that ND nanozymes could be used as ideal PTAs for photothermal sterilization. To study the photothermal performance, the ND nanozymes with gradient concentrations were irradiated by the NIR laser (808 nm, 0.75 W/cm^2^) for 10 min (Figure 2B). Particularly, when the concentration of ND nanozymes was ∼75 μg/mL, the temperature could be increased from 28 to 51.4 °C, whereas the temperature of pure water only increased by 1.6 °C under the same condition (Figure 2C). The photothermal effect of ND nanozymes exhibited an irradiation time-dependent, concentration-dependent, and power density-dependent elevation, like that of most reported PTAs (Figures 2D and S3–S6). The photothermal conversion efficiency of ND nanozymes was measured to be ∼43.8% based on the previously reported method (Figures 2E and 2F) (Wang et al., 2019b), which was comparable to those of many reported PTAs (Table S1) (Yong et al., 2014). In addition, the photothermal stability of ND nanozymes was explored by six laser irradiation cycles, the amplitude of temperature elevation, the UV-visible-NIR (UV-vis-NIR) absorption spectrum, and the color of ND nanozyme solution were not significantly changed before and after cycling laser irradiation (Figures 2G, 2H, and S7), suggesting that the as-obtained ND nanozymes possessed satisfactory photothermal stability. Hence, the prominent photothermal performance of ND nanozymes suggested that they could be used as potential PTAs for photothermal sterilization.

### Catalytic Properties and GSH-Depleting Functions of ND Nanozymes

**Figure 3:**
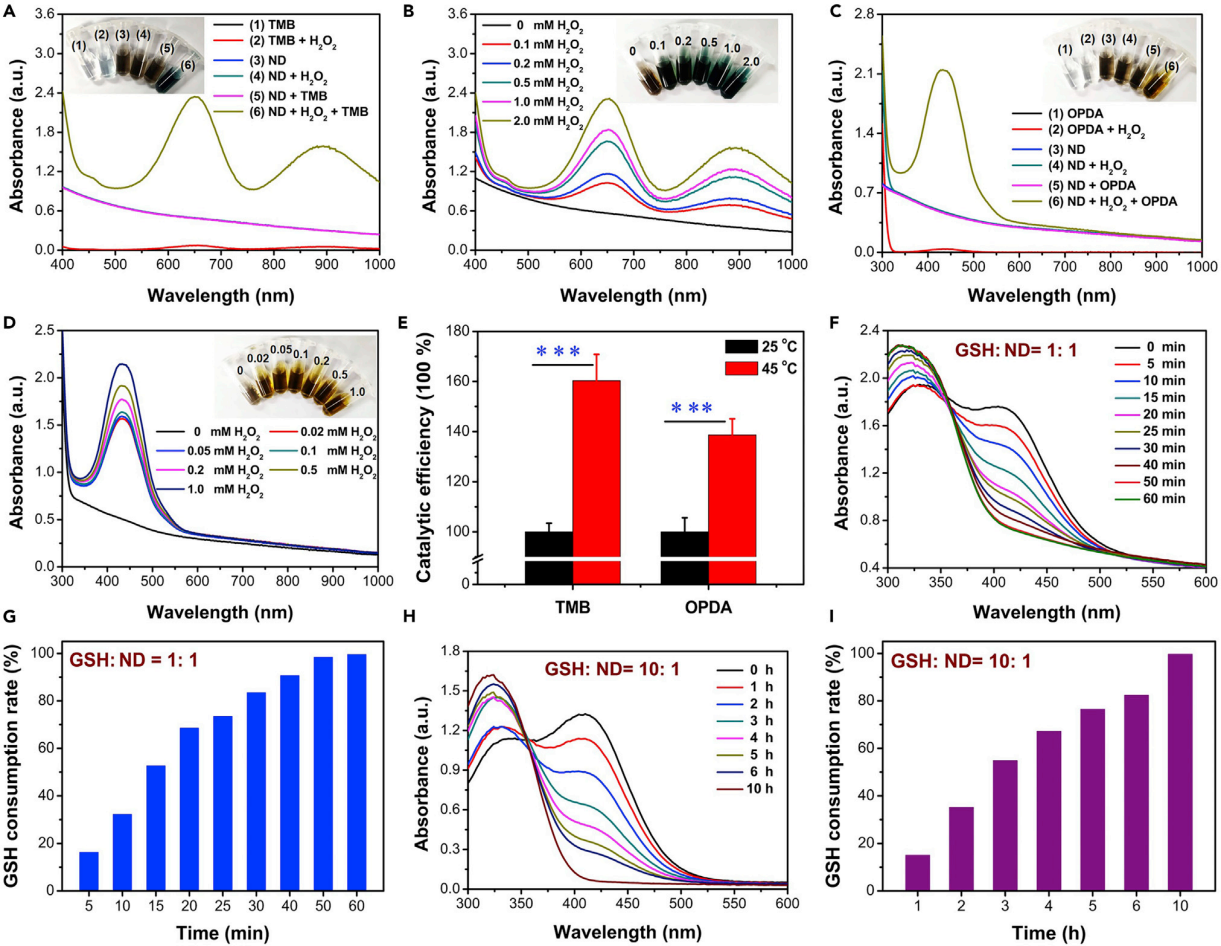
Catalytic Properties and GSH-Depleting Functions of ND Nanozymes (A and B) (A) The peroxidase-like catalytic effect of ND nanozymes at different conditions and (B) at various H_2_O_2_ concentrations by using the TMB probe. (C and D) (C) The peroxidase-like catalytic effect of ND nanozymes at different conditions and (D) at various H_2_O_2_ concentrations by using the OPDA probe. (E) Effect of temperature on its catalytic activity. (F and G) (F) Time-dependent GSH depletion and (G) GSH consumption rate by ND nanozymes (the mole ratio of GSH and ND nanozymes is 1: 1). (H and I) (H) Time-dependent GSH depletion and (I) GSH consumption rate by ND nanozymes (the mole ratio of GSH and ND nanozymes is 10: 1). See also Figure S8.

Nickel-based compounds are ideal catalysts with the properties of HRP-like enzyme, which have been reported for the detection of H_2_O_2_ and glucose. However, their catalytic properties have not been reported in the field of nanomedicine. The excellent catalytic activity of nickel-based compounds makes them have broad application prospects in sterilization. The HRP-like catalytic properties of the synthesized ND nanozymes were evaluated through the catalytic oxidation of 3,3′,5,5′-tetramethylbenzidine (TMB), one of the most widely used indicators of ⋅OH, which would be gradually changed from colorless to blue. In the presence of H_2_O_2_, the ND nanozymes changed the color of TMB probe from colorless to dark blue, whereas the control group showed no color change (Figure 3A). With the increase of H_2_O_2_ concentration, the color of the solution became bluer (Figure 3B), indicating that the catalytic activity of the ND nanozymes depended on the concentration of H_2_O_2_. Besides, o-phenylenediamine (OPDA) probe was further used to check the catalytic properties of ND nanozymes. Similar phenomena and results were observed by using the OPDA probe (Figures 3C–3E), which indicated that the ND nanozymes have excellent HRP-like enzyme catalytic properties. The Michaelis-Menten constant (K_m_) and maximum velocity (V_max_) of ND nanozymes were calculated by TMB detection, which were calculated to be ∼3.64 mM and ∼1.55×10^−4^ mM min^−1^, respectively (Figure S8). This relatively low Km value indicated the good catalytic performance of ND nanozymes.

Some reducing substances such as glutathione (GSH) present in bacteria (0.1–10 mmol/L) can weaken ROS-induced bacterial killing. Based on this, the properties of ND nanozymes in depleting GSH were explored by using 5,5′-dithio-bis (2-nitrobenzoic acid) (DTNB) probe (Shan et al., 2019). When the molar ratio of GSH to ND nanozymes was 1: 1, the characteristic peak of DTNB at 420 nm gradually decreased with the extension of reaction time (Figure 3F) and the GSH could be completely consumed after 1.0-h incubation, indicating that the ND nanozymes had a remarkable GSH consumption ability (Figure 3G). The clearance rate of GSH was ∼16.27%, 32.24%, 68.51%, 83.47%, 90.60%, and 99.58% at 5, 10, 20, 30, 40, and 60 min, respectively. When the molar ratio of GSH to ND nanozymes was increased to 10: 1, GSH still was completely consumed, but the rate of GSH consumption became slower (Figures 3H and 3I), indicating that the ability of ND nanozymes to consume GSH exhibited dose-dependent performance. The clearance rate of GSH was ∼15.03%, 35.10%, 54.83%, 67.15%, 82.36%, and 99.66% at 1, 2, 3, 4, 6, and 10 h, respectively. As the concentration of GSH was much higher than that of ND nanozymes, the ability of ND nanozymes to consume GSH could not be explained by redox. It was more likely that the ND nanozymes acted as GSH-like mimetic enzymes based on previous reports, resulting in extraordinary GSH-depleting function of ND nanozymes (Zhong et al., 2020).

### *In Vitro* Antibacterial Performance of ND Nanozymes

**Figure 4:**
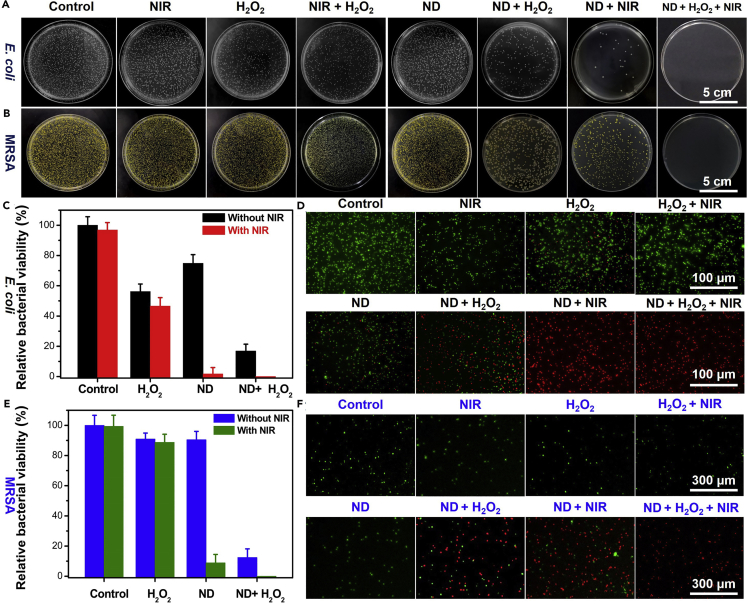
*In Vitro* Antibacterial Performance of the ND Nanozymes (A–F) Photographs of the bacterial colonies formed by (A) *E. coli* and (B) MRSA after different treatments. (C and E) Survival rates corresponding to (A) and (B). Fluorescent images of (D) *E. coli* and (F) MRSA stained with propidium iodide and SYTO 9 after different treatments.

Based on the excellent properties including photothermal effect, GSH-depleting function, and catalytic performance, it is speculated that the ND nanozymes could act as potential therapeutic agents for the treatment of bacterial infection by photothermal and catalytic properties. Therefore, the *in vitro* synergistic antibacterial effect of the ND nanozymes against *Escherichia coli* (*E. coli*, DH5α) and methicillin-resistant *Staphylococcus aureus* (MRSA, Mu50) was systematically evaluated. As for killing *E. coli*, lots of viable colonies formed on LB agar plates in the groups of control and NIR, suggesting that NIR irradiation alone did not affect *E. coli* growth. The antibacterial effect of the groups of H_2_O_2_ and H_2_O_2_ + NIR was weak, which was caused by the poor bactericidal effect of the limited H_2_O_2_. Interestingly, the pure ND nanozymes themselves also had some antibacterial effect, which may be due to their ability to attach on the surface of bacteria, resulting in the death of some bacteria. In the group of ND nanozymes + H_2_O_2_, the more obvious antibacterial efficiency was observed compared with the groups of H_2_O_2_ and ND nanozymes, with the bacterial survival rate of ∼16.87%. These data demonstrated that the ND nanozymes could catalyze the degradation of H_2_O_2_ by Fenton-like reaction and produce more toxic ⋅OH, which further enhanced the bactericidal effect. In the group of ND nanozymes + NIR, the survival rate of bacteria was only ∼1.67%, suggesting that the photothermal antibacterial effect of ND nanozymes had good results. In the group ND nanozymes + H_2_O_2_ + NIR, the bacteria had completely died, and the inhibition rate was ∼100% (Figures 4A and 4C). Similarly, as for killing MRSA, the corresponding treatment groups showed the same antibacterial trend as *E. coli* (Figures 4B and 4E). All these data together demonstrated that the photothermal-catalytic antibacterial effect of ND nanozymes had achieved more obvious sterilization efficiency.

To further clearly evaluate the antibacterial effect of ND nanozymes, the bacterial samples (*E. coli* and MRSA) after different treatments were immediately stained with SYTO 9 (viable bacteria, green fluorescence) and propidium iodide (dead bacteria, red fluorescence) for 30 min, respectively, and then were observed with a fluorescence microscope. In the control, NIR, H_2_O_2_, H_2_O_2_ + NIR, and ND nanozymes groups, only negligible red fluorescence was observed in *E. coli* and MRSA. However, a large amount of red fluorescence was observed in the ND nanozymes + H_2_O_2_ group, indicating that the ⋅OH generated by the Fenton-like reaction could significantly increase the antibacterial effect. In addition, a large amount of red fluorescence was observed in the ND nanozymes + NIR group, whereas only red fluorescence was found in the ND nanozymes + H_2_O_2_ + NIR group (Figures 4D and 4F). These results indicated that the combination of photothermal performance and catalytic properties of ND nanozymes could significantly improve its bactericidal efficiency.

**Figure 5:**
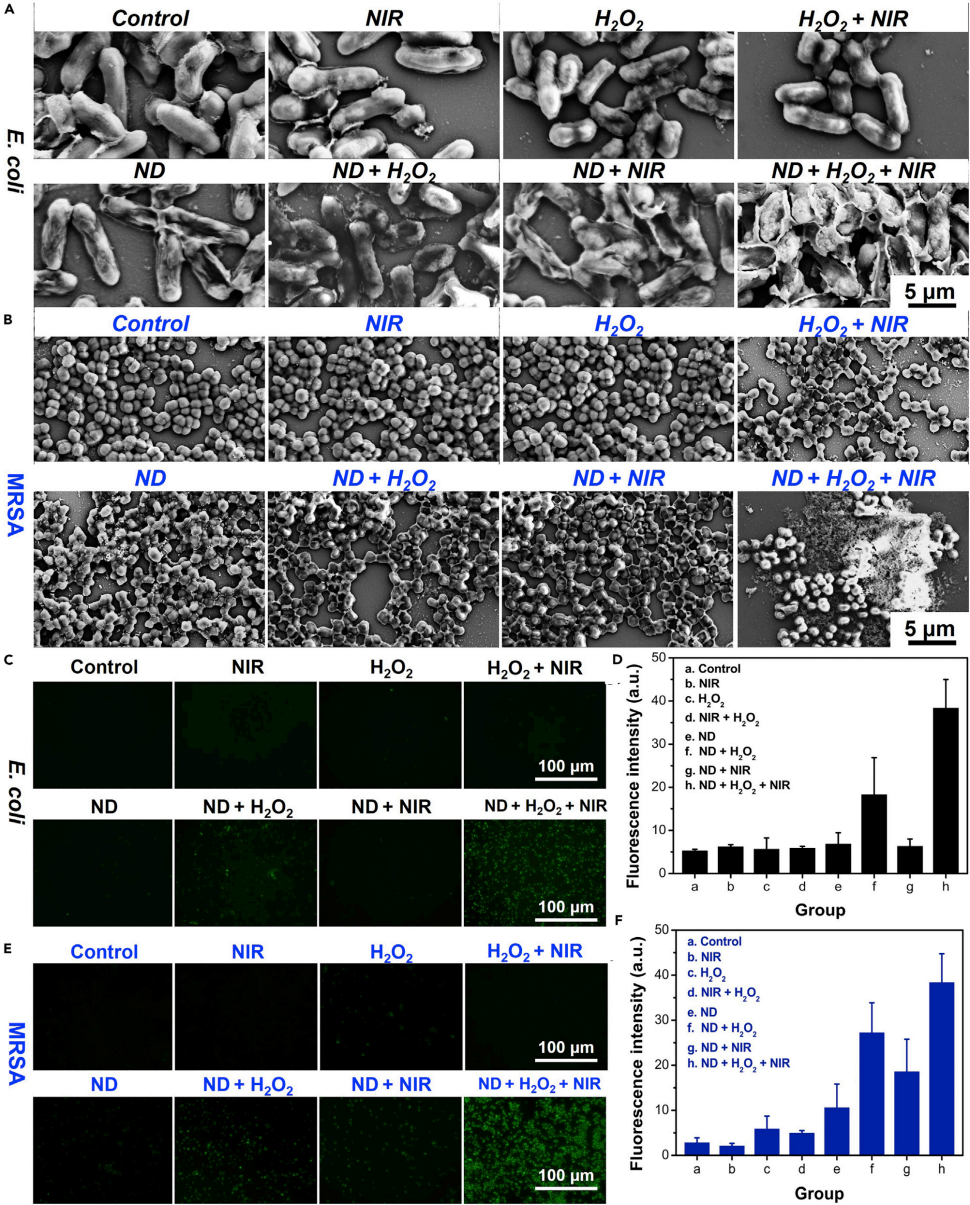
Antibacterial Mechanism of ND Nanozymes (A–F) SEM images of the (A) *E. coli* and (B) MRSA samples after different treatments. The fluorescent images of (C) *E. coli* and (E) MRSA stained with 2′,7′-dichlorodihydrofluorescein diacetate after different treatments; (D) and (F) the relative fluorescence intensities corresponding to (C) and (E), respectively.

From the aforementioned study, the ND nanozymes achieved good bactericidal effect *in vitro*, and then we further evaluated its antibacterial mechanism. Scanning electron microscopy (SEM) was used to elucidate the antibacterial effect by observing the morphological changes of bacteria from different groups (Figures 5A and 5B). The surfaces of bacteria (*E. coli* and MRSA) were intact and smooth in the control group, similarly, the bacteria in the NIR, H_2_O_2_, H_2_O_2_ + NIR, and ND nanozymes groups also showed negligible distortion and wrinkle, indicating that these groups had little effect on the integrity of bacterial cell membrane after different treatment. On the contrary, there were obvious cell deformation and content leakage in the ND nanozymes + H_2_O_2_ group, which indicated that the catalytic performance of ND nanozymes caused the bacterial damage. However, the bacterial surfaces became more wrinkled and rougher in the ND nanozymes + NIR group compared with ND nanozymes + H_2_O_2_ group, while the bacteria completely lost their cell integrity and the matrix flowed out, which implied that the synergistic effect had a stronger antibacterial ability. Therefore, this novel antibacterial nanoagent with excellent photothermal performance and catalytic effect based on ND nanozymes could rapidly and effectively destroy *E. coli* and MRSA.

It is well known that oxidative stress plays an important role in the nanomaterials-mediated antimicrobial systems (Yin et al., 2016). The first thing that needs to be known for this antibacterial strategy is whether ROS-dependent oxidative stress will occur. First, the ROS probe, 2′,7′-dichlorodihydrofluorescein diacetate was used to detect ROS after different treatments (Wang et al., 2019b). No obvious green fluorescence was observed in the control, NIR, H_2_O_2_, H_2_O_2_ + NIR, ND nanozymes, and ND nanozymes + NIR groups, but weak green fluorescence was observed in ND nanozymes + H_2_O_2_ group, indicating that ND nanozymes could effectively generate ⋅OH in the presence of H_2_O_2_. More importantly, the strongest green fluorescence was found in the ND nanozymes + H_2_O_2_ + NIR group, compared with other groups (Figures 5C–5F), indicating that the heat generated by PTT further improved the catalytic performance of ND nanozymes, thus producing more ⋅OH and leading to better bactericidal effect.

**Figure 6:**
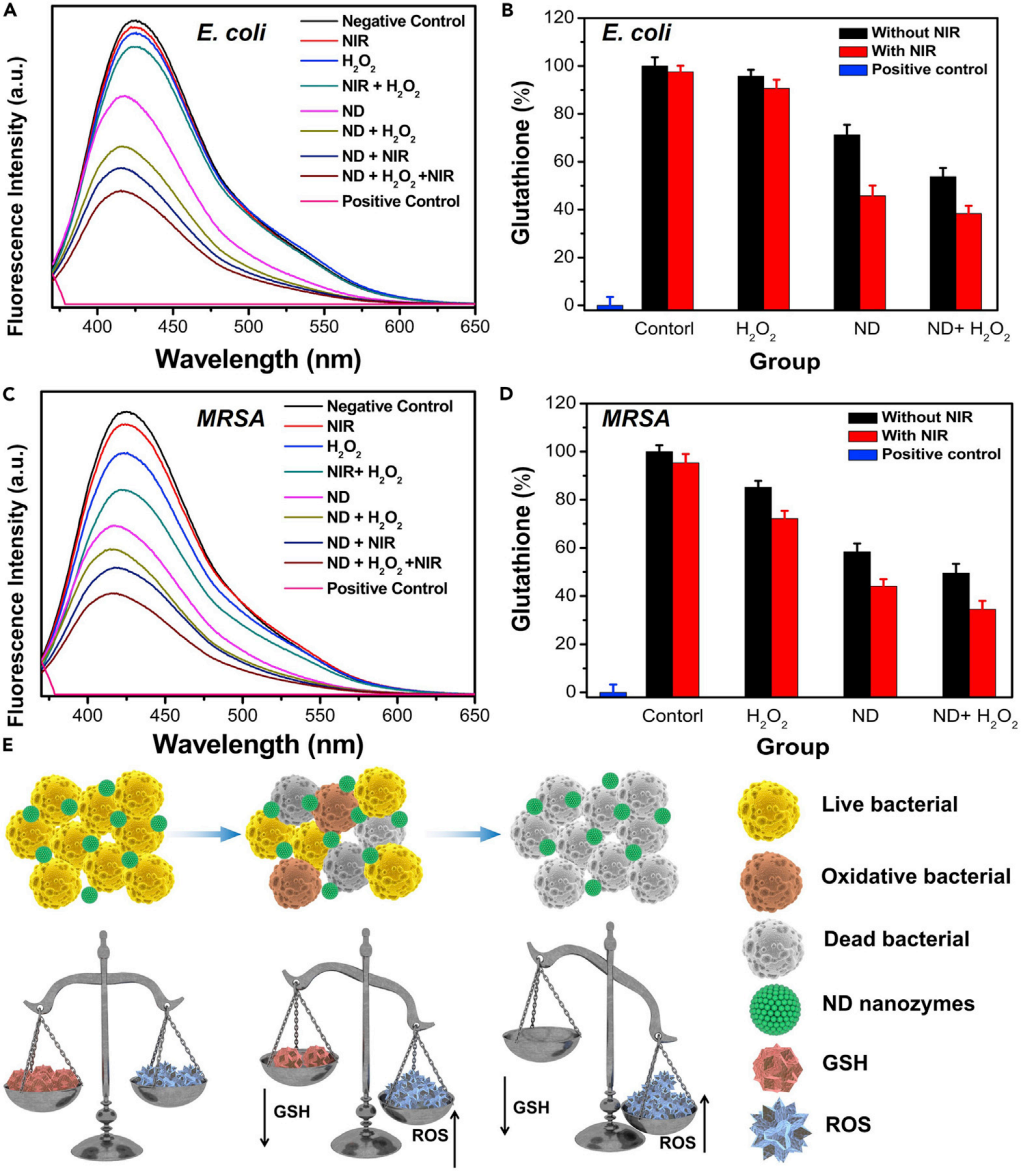
GSH Depletion of ND Nanozymes (A–E) (A) Fluorescence spectra and (B) percentage of GSH depletion of *E. coli* after different treatments; (C) fluorescence spectra and (D) the percentage of GSH consumption of MRSA after different treatments; (E) schematic diagram of GSH depletion by the ND nanozymes on sterilization.

Second, GSH is a tripeptide molecule widely existing in bacteria (0.1–10 mmol/L) that played an important role in the antioxidant defense system of bacteria and effectively prevented oxidative stress from damaging cell components (De Zoysa et al., 2008; Yan et al., 2013). Therefore, GSH level can be used as an indicator of bacterial oxidative stress, and the GSH depletion by the ND nanozymes was determined via the Ellman method. In the experiment, the fluorescence probe o-phthaldialdehyde was used to detect intracellular GSH changes. For *E. coli*, GSH could not be obviously consumed in the control (percentage of GSH consumption: 0% ± 3.64%), H_2_O_2_ (4.28% ± 2.66%), NIR (2.75% ± 2.55%), and H_2_O_2_ + NIR (9.37% ± 3.62%) groups, and it could be found that ND nanozymes (28.81% ± 4.25%) could effectively deplete GSH, further indicating that the ND nanozymes had good GSH depletion ability. GSH consumption was further improved in the ND nanozymes + H_2_O_2_ (46.23% ± 3.67%) and ND nanozymes + NIR (54.25% ± 4.32%) groups, whereas in the ND nanozymes + H_2_O_2_ + NIR (61.68% ± 3.24%) group the reduction of GSH content in bacteria was observed to be the most significant (Figures 6A and 6B). Similarly, the same experimental phenomena and results could be observed in MRSA bacteria (Figures 6C and 6D), further indicating that ND nanozymes could effectively consume GSH in bacteria, thereby breaking the balance of bacterial homeostasis and significantly improving sterilization effect.

The possible antibacterial mechanism of ND nanozymes was proposed based on the aforementioned results. (1) The ⋅OH produced by H_2_O_2_ caused cell wall damage in the presence of ND nanozymes. (2) The mild photothermal performance could further enhance the catalytic activity of ND nanozymes. (3) The destructive physical interaction of bacteria with the sharp edges of ND nanozymes was enhanced due to increased component motion at high temperatures. (4) The ND nanozymes themselves could consume GSH in bacteria, which effectively disrupted the internal balance of the internal environment of bacteria, leading to its death (Figure 6E). Collectively, the different antibacterial mechanisms, namely, the inherent antibacterial capacity (ROS production and GSH consumption, etc.) and high photothermal sterilization, were the main reasons for its high antibacterial performance.

### *In Vivo* Antibacterial Activity Assessment of ND Nanozymes

**Figure 7:**
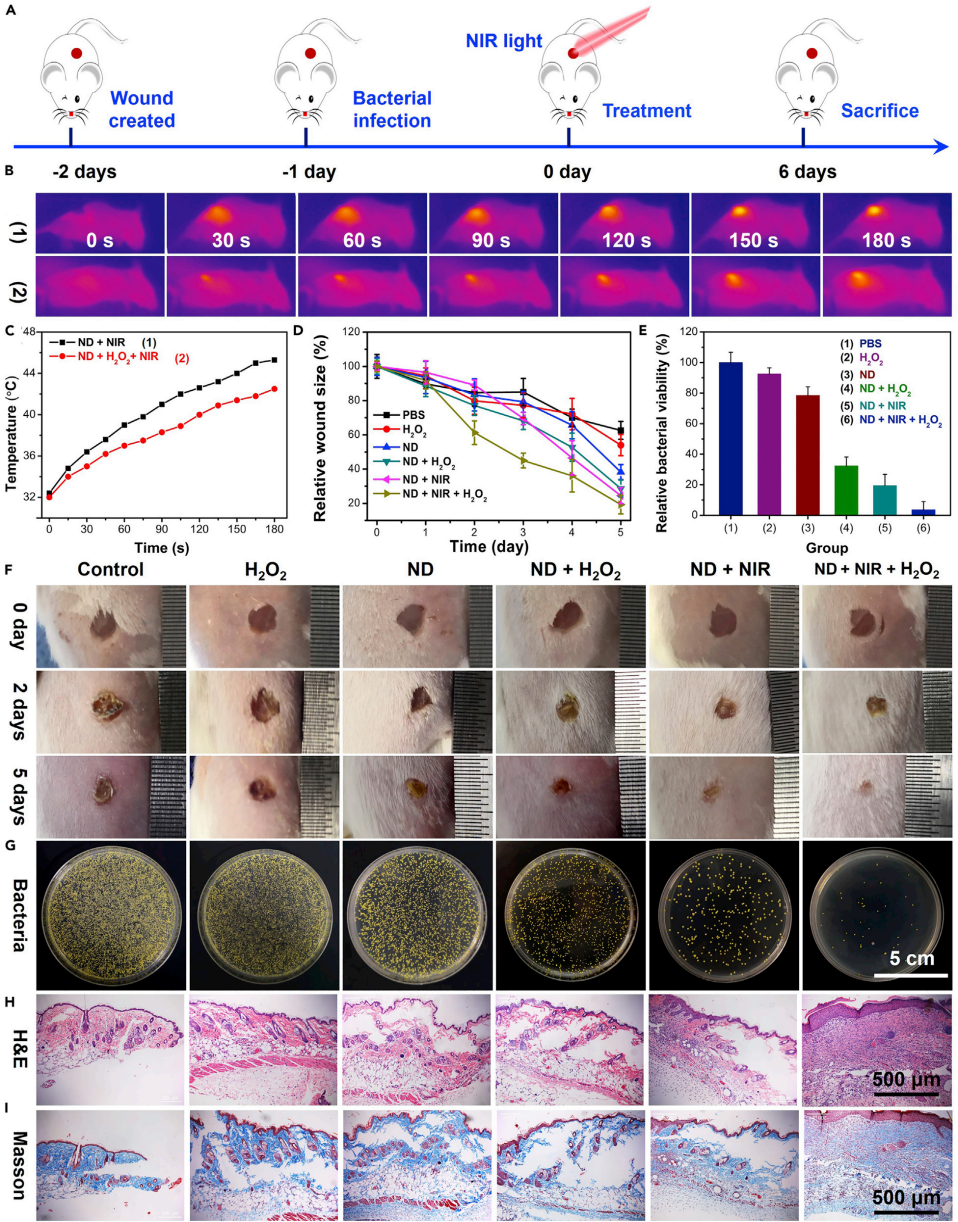
*In Vivo* Antibacterial Activity Assessment of the ND Nanozymes (A–I) (A) *In vivo* antibacterial protocol in mice. (B) Infrared images and (C) temperature-elevating curves of the localized bacterial infection sites treated with ND nanozymes under NIR irradiation (808 nm, 1.0 W/cm^2^, 2 min). (D) The relative wound size and (F) the corresponding digital photographs of mice in MRSA-infected group (n = 5). (E) The numbers of viable MRSA in infected wounds after different treatments determined by the plate counting method. (G) The photographs of bacterial colonies formed on LB agar plates from the infected wounds after different treatments. The photomicrographs of tissue sections of MRSA-infected wounds in mice after different treatments by using (H) H&E and (I) Masson staining. See also Figures S9 and S10.

Inspired by the excellent antibacterial effects and biosafety *in vitro*, we further evaluated the antibacterial properties of ND nanozymes *in vivo* by using the MRSA-infected wound model (Figure 7A). First, female BALB/c mice were randomly divided into the following six groups (n = 5): (1) PBS, (2) H_2_O_2_, (3) ND nanozymes, (4) ND nanozymes + H_2_O_2_, (5) ND nanozymes + NIR light, and (6) ND nanozymes + H_2_O_2_ + NIR light (808 nm, 1.0 W/cm^2^, 3 min). And MRSA, as a common pathogen of skin infection, was used to build a wound healing model. The concentrations of ND nanozymes and H_2_O_2_ were 50 μg/mL and 0.2 mM, respectively. The temperature of the wound was monitored by an infrared thermal camera (Figure 7B). The results showed that the temperature of the wound changed significantly under laser irradiation (808 nm, 1.0 W/cm^2^, 3 min) in the ND nanozymes + NIR light and ND nanozymes + H_2_O_2_ + NIR light groups, but there was no significant change in the control group (Figure 7C). It should be noted that the temperature of the wound should be controlled around 45 °C to reflect the synergistic treatment effect of ND nanozymes. There was no significant difference in ND nanozymes and H_2_O_2_ alone compared with the control group after 4 days. In the ND nanozymes + H_2_O_2_, ND nanozymes + NIR light, and ND nanozymes + H_2_O_2_ + NIR light groups, the wounds showed remarkable healing and size reduction, and their relative wound area was reduced to ∼28.65%, ∼24.50%, and ∼19.25%, respectively (Figures 7D and 7F). Furthermore, the antibacterial effects of different treatments *in vivo* were evaluated. Compared with the control group, the relative percentage of live bacteria in the wounds in the groups of ND nanozymes + H_2_O_2_ and ND nanozymes + NIR light was ∼32.37% and ∼19.47%, respectively. However, the relative percentage of live bacteria in the wounds was only ∼3.61% in the ND nanozymes + H_2_O_2_ + NIR light group, which was significantly lower than those of the other five groups (Figures 7E and 7G). The results showed that the photothermal and catalytic properties of ND nanozymes alone had a good effect on sterilization, but the wound healing was the most obvious for the combination therapy, indicating that rapid and effective sterilization using the synergistic effect of catalytic treatment and PTT played an important role in the wound healing.

The effect of different treatments on wound healing was further evaluated by histological analysis. H&E staining showed that intact epidermal layers of wound tissue were only seen in the ND nanozymes + H_2_O_2_ + NIR light group, whereas more inflammatory cells were found in other five groups (Figure 7H). In addition, the presence of hair follicles in this group also indicated that the wound healed well. Furthermore, the collagen deposition of the wound tissues was evaluated by the Masson's trichrome staining. In the skin tissue treated with ND nanozymes + H_2_O_2_ + NIR, the collagen fibers stained blue were continuous, which was more obvious than the control group and the simple treatment group, indicating that the photothermal-catalytic combination therapy had better recovery (Figure 7I). Therefore, combined with the excellent catalytic performance and photothermal effect of ND nanozymes, effective antibacterial treatment of infectious wounds was successfully demonstrated. In addition, within the 5-day anti-infection period, there was no significant difference in body weight between mice in each group and no significant health abnormalities occurred (Figure S9). From the H&E images of the main organs of the infected or treated mice, no substantial pathological damage and inflammation were observed during the treatment (Figure S10), indicating that the as-prepared anti-infective ND nanozymes had outstanding biocompatibility.

### Biodegradable Behavior and Biosafety Evaluation of ND Nanozymes

**Figure 8:**
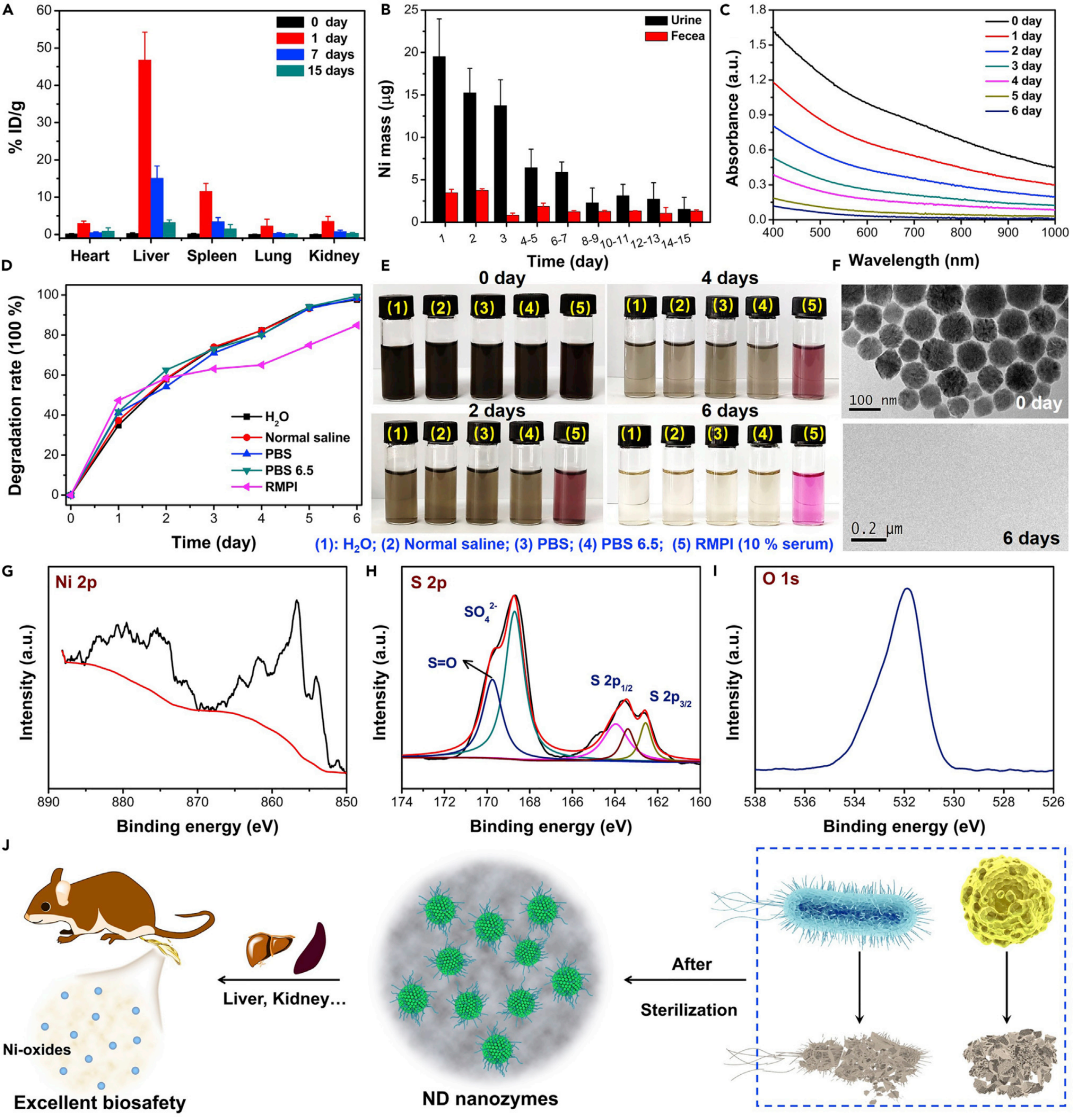
*In Vitro* and *In Vivo* Degradation Behavior of ND Nanozymes (A–J) (A) Time-dependent biodistribution and (B) excretion patterns at different time points post i.v. injection with the ND nanozymes (dose: 10 mg/kg). (C) UV-vis-NIR absorption spectra of ND nanozymes in pure water over time. (D) The degradation rate of ND nanozymes in different physiological solutions (pure water, normal saline, PBS, PBS pH = 6.5, RPMI containing 10% serum) on the sixth day. (E) Photographs of ND nanozymes in physiological solutions at various time points. (F) TEM images of ND nanozymes in pure water on the 0 and fourth day. (G–I) XPS spectra of ND nanozymes in pure water on the fourth day; core level spectra of (G) Ni 2p, (H) S 2p, and (I) O 1s. (J) The degradation mechanism of ND nanozymes *in vivo*. See also Figures S11 and S12.

Biodegradable nanomaterials have higher safety than non-metabolizable nanomaterials and thus have received extensive attention in the biomedical field (Yang et al., 2019b). Many metal chalcogenides have been shown to be biodegradable nanomaterials, and it is speculated that ND nanozymes may also be a novel biodegradable nanomaterial (He et al., 2016; Wang et al., 2020b). To determine whether the synthesized ND nanozymes could be biodegraded, we carefully investigated the biodistribution and metabolic pathways of the ND nanozymes. When the ND nanozymes were intravenously (i.v.) injected into mice, the biodistribution of ND nanozymes in the main organs (heart, liver, spleen, lung, and kidney) at different time points (0, 1, 7, and 15 days) was measured. On the first day, Ni element was mainly distributed in the liver (∼46.76% ID/g, the percent of injected dose per gram of tissue) and spleen (∼11.55% ID/g) of the mice, the content of Ni in the main organs of mice had become very low after 15 days, and the total of liver and spleen was measured to be ∼4.7% ID/g (Figure 8A), indicating that most of the ND nanozymes could be rapidly excreted from the body. It was worth noting that although ND nanozymes were gradually degraded in physiological environments and *in vivo*, however, it still showed relatively long blood circulation time after i.v. injection (Figure S11). More importantly, high concentrations of Ni element were detected in the feces and urine of mice on different days (Figure 8B), indicating that part of the ND nanozymes could be rapidly metabolized by the renal and fecal pathways.

To clarify the degradation and clearance mechanism of the synthesized ND nanozymes, a series of experiments were designed to prove our conjecture. With the prolongation of time, the UV-vis-NIR absorption of ND nanozymes aqueous solution gradually decreased, and the corresponding absorption at 808 nm was already very low on the sixth day (Figure 8C), suggesting that ND nanozymes could be rapidly degraded *in vitro*. Similar phenomena were also observed in other physiological solutions (H_2_O, normal saline, PBS, PBS pH = 6.5, RPMI 1640). More importantly, the degradation rate of ND nanozymes in these physiological solutions was above ∼90% on the 6^th^ day (Figures 8D and S12). In addition, the color of ND nanozymes in these solutions gradually changed from black to colorless with extension of time (Figure 8E), which further proved that ND nanozymes could be rapidly degraded *in vitro*.

To further understand the changes in the composition and structure of ND nanozymes, the state of the sample was investigated by TEM and XPS on the fourth day. The newly prepared ND nanozymes exhibited a uniform spherical morphology, and no obvious structure was observed on the fourth day (Figure 8F). In addition, XPS was used to analyze the surface valence state change of the samples, and the characteristic peaks of Ni 2p and S 2p were significantly lower than those in the initial ND nanozymes (Figures 8G and 8H). Furthermore, obvious characteristic peaks of SO_4_^2−^, S-O bonds, O 1s were detected in the corresponding spectrum, which indicated that ND nanozymes were converted to nickel oxides (Figures 8H and 8I). Based on this, the ND nanozymes could be rapidly metabolized by the renal and fecal pathways, and the degradation mechanism of ND nanozymes could be mainly explained by the surface oxidation (Figure 8J). However, the detailed mechanism of bio-degradation needs further investigation.

**Figure 9:**
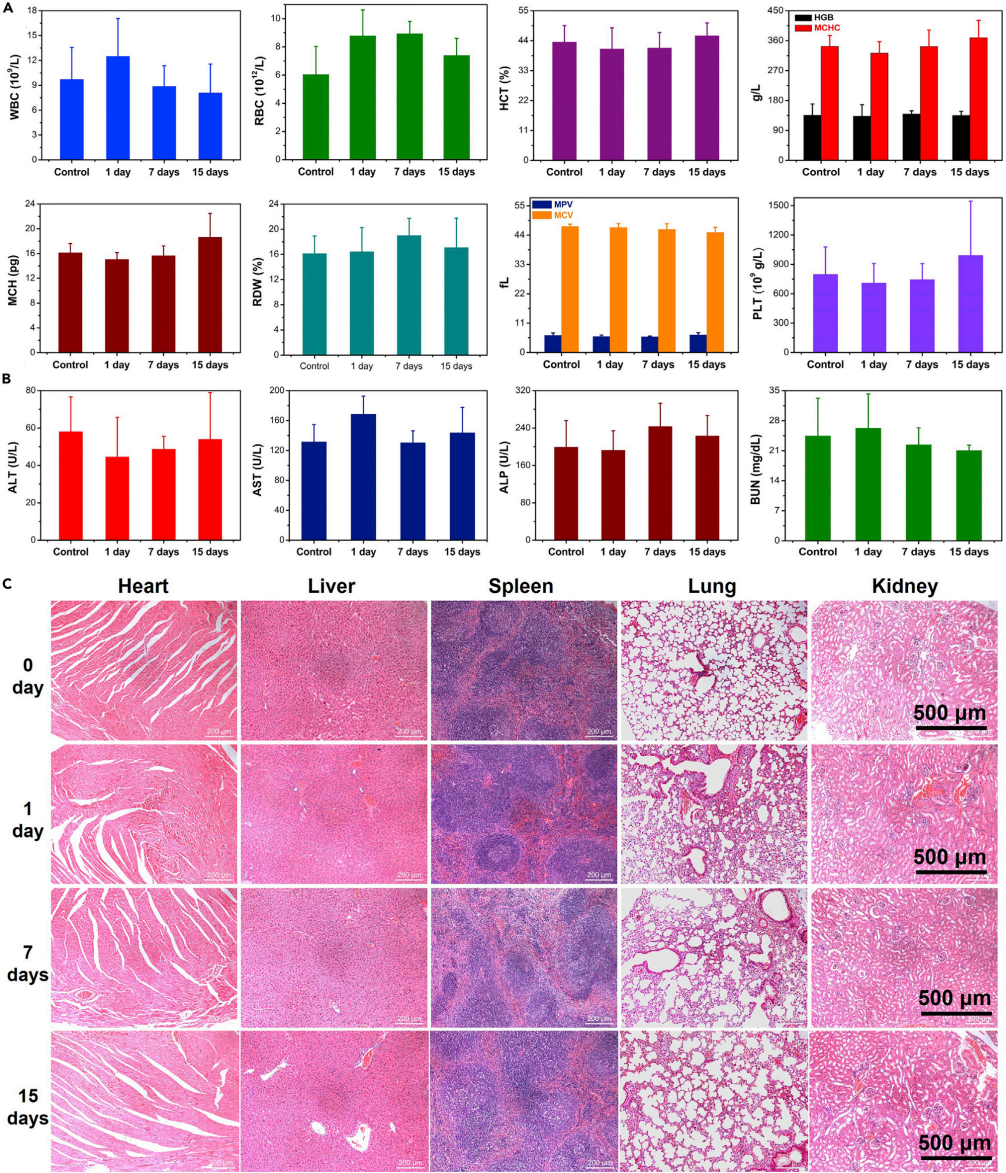
Biosafety Evaluation of the ND Nanozymes (A–C) (A) The blood panel analysis and (B) the blood biochemistry test. (C) H&E staining images of major organs (including heart, liver, spleen, lung, and kidney) of the mice after i.v. injection of ND nanozymes at different days (dose: 10 mg/kg). See also Figure S13.

Good biosafety is the primary prerequisite of nanomaterials used in the biomedical field, the biosafety of ND nanozymes was also studied in detail (Yan et al., 2019). The cytotoxicity of ND nanozymes *in vitro* was first evaluated by the typical MTT method, ND nanozymes exhibited no significant cytotoxicity even when the concentration reached 100 μg/mL (Figure S13). After i.v. injection of ND nanozymes at different time points, blood routine and blood biochemical data were collected from the mice. The parameters concerning the blood panel counts and serum biochemistry had no meaningful changes compared with the control group (Figures 9A and 9B), indicating that the ND nanozymes did not cause any significant toxicity with reasonable dose. In addition, the main organs (including heart, liver, spleen, lung, and kidney) were collected for H&E staining (Figure 9C). No obvious inflammation and edema were observed in these main organs, indicating that there was no obvious tissue damage and the satisfactory histocompatibility. Considering the rapid *in vivo* degradation of ND nanozymes, it is possible to conclude that there is no long-term toxicity of the biodegradable ND nanozymes, which highlights their enormous potential for clinical bacterial application.

## Discussion

In summary, monodisperse ND nanozymes were prepared through a simple solvothermal method based on the La Mer scheme, which have satisfactory photothermal performance, high photothermal conversion efficiency, excellent peroxide-like catalytic activity, and GSH-depleting function. The as-obtained ND nanozymes as HRP-like nanozymes could generate ⋅OH in the presence of H_2_O_2_ through Fenton-like reaction, and the catalytic activity of Fenton-like reaction was further enhanced by the mild photothermal performance generated by PTT, which led to the achievement of photothermall-enhanced catalytic bacterial treatment *in vitro*. More importantly, the experimental results of wound healing *in vivo* displayed that the synergetic antimicrobials could be used to disinfect wounds. Moreover, the ND nanozymes acted as GSH-like mimetic enzymes, thus possessing prominent GSH-depleting function, which could effectively consume some reducing substances in bacteria, resulting in better antibacterial effects. Interestingly, these novel ND nanozymes owing to their rapid degradation properties could be rapidly metabolized by the renal and fecal pathways, without causing any significant toxicity. Collectively, the ND nanozymes as biodegradable multifunctional antibacterial agents have a wide application prospect in precise sterilization.

### Limitations of the Study

Although biodegradable ND nanozymes achieved significant synergistic antibacterial effect *in vitro* and *in vivo*, their synergistic mechanism of antibacterial treatment and the biodegradable mechanism still need to be further explored.

### Resource Availability

#### Lead Contact

Further information and requests for resources and reagents should be directed to and will be fulfilled by the Lead Contact, Prof. Liang Cheng (lcheng2@suda.edu.cn).

#### Materials Availability

This study did not generate new unique reagents.

#### Data and Code Availability

This study did not generate datasets/code.

## Methods

All methods can be found in the accompanying Transparent Methods supplemental file.

## Supplemental Information

Document S1. Transparent Methods, Figures S1–S13, and Table S1
