# Supplementary material for: Biodegradable Nickel Disulfide Nanozymes with GSH-Depleting Function for High-Efficiency Photothermal-Catalytic Antibacterial Therapy
Source: iScience. 2020 Jun 16;23(7):101281. doi: 10.1016/j.isci.2020.101281 (PMC7334425; doi:10.1016/j.isci.2020.101281)
Supplement: Document S1. Transparent Methods, Figures S1–S13, and Table S1 [file mmc1.pdf]

iScience, Volume *23*

Supplemental Information

Biodegradable Nickel Disulfide Nanozymes with GSH-Depleting Function for High-Efficiency Photothermal-Catalytic Antibacterial Therapy

Xianwen Wang, Linxin Fan, Liang Cheng, Yanbin Sun, Xiyu Wang, Xiaoyan Zhong, Qianqian Shi, Fei Gong, Yu Yang, Yan Ma, Zhaohua Miao, and Zhengbao Zha

Supplementary information

Biodegradable nickel disulfide nanozymes with GSH-depleted function for high-efficiency photothermal-catalytic antibacterial therapy

Xianwen Wang[1, 2], Linxin Fan[1], Liang Cheng[2, 4]*, Yanbin Sun[1], Xiyu Wang[2], Xiaoyan Zhong[3], Qianqian Shi[1], Fei Gong[2], Yu Yang[2], Yan Ma[1], Zhaohua Miao[1], and Zhengbao Zha[1]*

[1]School of Food and Biological Engineering, Hefei University of Technology, Hefei, 230009, P. R. China.

[2]Institute of Functional Nano & Soft Materials (FUNSOM), Jiangsu Key Laboratory for Carbon-based Functional Materials and Devices, Soochow University, Suzhou 215123, P. R. China

[3]National Engineering Research Centre for Nanomedicine, College of Life Science and Technology, Huazhong University of Science and Technology, Wuhan 430074, P. R. China.

[4]Lead Contact

Corresponding Author:

***E-mail:** zbzha@hfut.edu.cn (Z. Zha), Phone: +86-551-62901285

***E-mail:** lcheng2@suda.edu.cn (L. Cheng), Phone: +86-512-65880927 (Lead Contact)

Transparent Methods

Materials and reagents: Polyvinylpyrrolidone (PVP, K30, MW=40000), hydrogen peroxide (H_2O_2, 30 wt%), and sodium thiosulfate pentahydrate ($Na_2S_2O_3$•$5H_2O$) were obtained from Sinopharm Chemical Reagent Co., Ltd. Nickel nitrate hexahydrate ($Ni(NO_3)_2$•$6H_2O$) was purchased from Aladdin Chemical Reagent Co., Ltd. 3-(4,5-dimethyl-2-thiazolyl)-2,5-diphenyl-2-Htetrazolium bromide (MTT), O-phenylenediamine (OPDA), 5, 5'-dithio-bis (2-nitrobenzoic acid) (DTNB), and 3, 3', 5, 5'-Tetramethylbenzidine (TMB) were bought from J&K Chemical Co., Ltd. All reagents were purchased from commercial sources and used without any further purification.

Bacteria: Escherichia coli (E. coli, DH5α) and methicillin-resistant staphylococcus aureus (MRSA, Mu50). The reason for choosing these two bacteria for the following reason: E. coli and MRSA are representative of Gram positive and negative bacteria, and the separation rate of these two bacteria is the best among the positive and negative bacteria, respectively. These two kinds of bacteria are easy to be separated and their purity are high. E. coli (DH5α) is a mutagenic strain, which mainly shows a lack of immunity to exogenous DNA, and it doesn't emerge drug resistance. Therefore, it can be used as a typical representative for studying non-resistant bacteria. MRSA, as a relatively common drug-resistant bacteria, has a high pathogenic rate and multiple transmission routes, which can cause many diseases and pose a certain threat to people's health and safety. The mu50 strain was discovered earlier and was one of the first three bacteria to have DNA sequence completely. It is safe and controllable for use in studies, and it is not particularly pathogenic. Based on this, it is reasonable to choose these two bacteria based on these considerations.

Synthesis of ND nanozymes: ND nanozymes were prepared by a simple solvothermal method based on our previously reported strategy. (He et al., 2019) Briefly, 0.3 g PVP, 1.0 mmol $Ni(NO_3)_2$•$6H_2O$ (0.291 g), and 3.0 mmol $Na_2S_2O_3$•$5H_2O$ (0.745 g) were completely dissolved in a mixture of 20 mL of ultrapure water and 20 mL of ethylene glycol under vigorous magnetic stirring. Thereafter, the mixed solution was put into the Teflon-lined sealed autoclave and stewed at 180 ^{o}C for 12 h. The products were obtained through thorough centrifugation/washing cycles. Next, the as-obtained ND nanozymes were further dialyzed for 24 h to remove the unreacted materials, and the final products were stored at -4 ^{o}C protected by N_2 for further use.

Characterizations of ND nanozymes: Transmission electron microscope (TEM, JEM-2100F, Japan), energy-dispersive X-ray spectroscopy (EDS) (Tecnal G2 F20 U-Twin), and high-angle annular dark-field scanning TEM (HAAD-FSTEM) were obtained to characterize morphology, structure, and composition of ND nanozymes. The crystalline structure of ND nanozymes was performed by X-ray diffraction (XRD, Panalytical Empyrean). The surface chemistry composition and chemical states of ND nanozymes were determined by X-ray photoelectron spectroscopy (XPS, ESCALab 250Xi). UV–vis–NIR spectrophotometer (Genesys™ 10S UV–vis, Thermo Scientific) was used to measure UV-vis-NIR absorption spectra of samples. The photothermal data and thermal imaging pictures were recorded by using an IR thermal camera (Fortric 225).

Photothermal performance of ND nanozymes: To evaluate the photothermal effect of ND nanozymes, 1.0 mL aqueous solution of ND nanozymes with different concentrations (0-75 μg/mL) were added in quartz cells and irradiated by 808 nm laser with different power densities (0.2-1.5 W/cm^2) for 10 min, respectively. The photothermal stability of ND nanozymes was determined by six laser on/off cycles irradiation, and the photothermal conversion efficiency of ND nanozymes was calculated using the methods previously described.(Wang et al., 2019b)

Peroxides-like catalytic activity of ND nanozymes: The •OH was analyzed according to the principle that •OH could oxide TMB or OPDA probe and strengthen the absorbance of TMB at 664 nm or OPDA at 414 nm.(Wang et al., 2020) Briefly, the TMB and OPDA were used as the indicators to visualize and monitor the •OH generation. In detail, 1.0 mL acetate buffer containing ND nanozymes (20 μg/mL), TMB (0.5 mM), and H_2O_2 with different concentrations (0-1.0 mM) was incubated for 30 min at room temperature (25 ^oC). The color of the solution was imaged and the UV-vis-NIR spectra was measured. The catalytic performance of ND nanozymes at 45 ^oC was further explored to simulate the effect of catalytic property by the mild photothermal treatment.

Calculation of the Michaelis–Menten constant (K_M) and maximum velocity (V_{max}): Results based on TMB tests were used to calculate K_M and V_{max}, all average initial velocities of absorbance changes would then be converted as initial velocities (v_0) of cation-free radical production or hydroxyl radical formation via the Beer Lambert law **(Eq. 1)**, which were then plotted against the corresponding

concentration and fitted with Michaelis Menten curves **(Figure S8a) (Eq. 2)**. Moreover, to determine the K_M and V_{max}, a linear double-reciprocal plot **(Lineweaver Burk plot, Eq. 3)** was obtained as given in **Figure S8b**. According to the calculation, the K_M and V_{max} values were calculated to be 3.64 mM and 1.55×10^{-4} mM min^{-1} for ND nanozymes.

$$A = kbc \quad \textbf{(Eq. 1)}$$

$$v_0 = \frac{V_{max} \cdot [S]}{K_m + [S]} \quad \textbf{(Eq. 2)}$$

$$\frac{1}{v_0} = \frac{K_m}{V_{max}} \cdot \frac{1}{[S]} + \frac{1}{V_{max}} \quad \textbf{(Eq. 3)}$$

GSH-depleted property of ND nanozymes: The consumption of GSH was detected with DTNB probe by UV–vis–NIR spectroscopy.(Wan et al., 2019) The as-obtained ND nanozymes with different concentrations (0.1 mM or 1.0 mM) were mixed with GSH (1.0 mM) at room temperature. At different points, 100 μL of the mixture was added into 900 μL phosphate buffer saline (PBS, pH 7.4), and then DTNB (0.1 mM) was added the mixed solution. After 2 min later, the absorbance spectrum of this mixed solution was recorded by UV–vis–NIR spectrophotometer.

***In vitro* cytotoxicity experiments:** Human umbilical vein endothelial cells (HUVECs) were used for evaluation the cytotoxicity of ND nanozymes. Briefly, the HUEVCs with the density of 1×10^4 cells for each well were seeded into 96-well plates for 24 h. Then the cells incubated with ND nanozymes with the varying concentrations (0-100 μg/mL) and cultured for another 12 h. Finally, the relative cell viabilities were determined using a standard MTT method. (Guan et al., 2019; Wang et al., 2019b)

***In vitro* antibacterial experiments:** During antibacterial investigations, Escherichia coli (*E. coli*, DH5α) was used as the model Gram (-) bacterial and methicillin-resistant staphylococcus aureus (MRSA, Mu50) as model Gram (+) bacterial cell strains. The strains were revived from frozen glycerol stocks by inoculation in Luria–Bertani (LB) medium overnight at 37 °C in a humidified incubator. The population density of the bacterial cells was determined by measuring the absorbance at 600 nm. In a typical antibacterial assay, *E. coli* or MRSA were divided into following eight groups: (1) bacteria; (2) bacteria + NIR light; (3) bacteria + H_2O_2; (4) bacteria + NIR light + H_2O_2; (5) bacteria + ND nanozymes; (6) bacteria + ND nanozymes + H_2O_2; (7) bacteria + ND nanozymes + NIR light; (8)

bacteria + ND nanozymes + H_2O_2 + NIR light. The groups of (2), (4), (7)-(8) were further exposed to NIR laser (808 nm, 1.0 W/cm^2) for 3 min. After the NIR irradiation, the procedure was the same as groups of (1), (3), (5)-(6). The final concentrations of ND nanozymes, H_2O_2, and bacteria were 100 μg/mL, 200 μM, and 1×10^6 colony forming units (CFU)/mL, respectively. The total volume of solution in each well was 0.5 mL. After incubation for 20 min, 100 μL of bacterial suspension of groups (1)-(8) were spread on the agar culture plate and incubated at 37 °C for 18 h, and the number of colonies was counted. All experiments were repeated for three times. The bacteria survival rate was calculated using the following equation: Survival viability (%) = $N_t/N_c \times 100$ %, where N_t represents the number of colonies formed in the experimental group and N_c refers to the number of colonies formed in the control group (PBS group). (Liu et al., 2019)

Live/dead staining test: The bacteria after various treatments stained with SYTO9 and propidium iodide (PI) for 30 min, followed by washing three times using PBS. Fluorescence microscopy was used to observe the live (green fluorescence) and dead (red fluorescence) bacterial by the fluorescence microscopy. (Miao et al., 2019)

ROS staining test: The method to study ROS generation in bacterial was tested using a modified 2', 7'-dichlorodihydrofluorescein diacetate (DCFH-DA) probe. The bacteria after different treatments stained with DCFH-DA (20 μM) for 30 min, followed by washing three times using PBS. After that, green fluorescence was observed using fluorescence microscope to prove the generation of ROS.

Morphological observation of the bacteria: Bacterial samples were centrifuged (4000 rpm, 3 min) and re-dispersed into 2.5% glutaraldehyde after respective treatments. After 12 h, the fixed samples were further dehydrated gradually using ethanol solution with increasing ethanol concentration (10%, 30%, 50%, 70%, and 90%) for 10 min each. Finally, the cell samples were dispersed in 200 μL ethanol and dipped on a carbon coated cooper grid for scanning electron microscope (SEM, FEI Quanta 200F) observation.

***In vivo* antibacterial activity and wound healing:** All the animal procedures were performed in compliance with the guidelines of the Institutional Animal Care and Use Committee of Department of Anhui Medical University (LLSC20150134). Mice were divided into following six groups randomly (n = 5): (1) control; (2) H_2O_2; (3) ND nanozymes; (4) ND nanozymes + H_2O_2; (5) ND nanozymes +

NIR light; (6) ND nanozymes + H_2O_2 + NIR light. These mice their right flanks shaved, followed by anesthetization with pentobarbital sodium. A skin wound ~ 100 mm^2 was created by nicking with sterile scalpel. Then, the MRSA suspension (50 μL, 1×10^9 CFU/mL) was gradually inoculated onto the wound and the moisture allowed to evaporate naturally. After 12 h, for the ND nanozymes + H_2O_2 + NIR light treatment group, 50 μL of ND nanozymes (100 μg/ml, dispersed into PBS) and 50 μL of H_2O_2 (200 μM, dispersed into PBS) were dropped onto the bacterial foci, then the wound was irradiated with an NIR laser (808 nm, 1.0 W/cm^2) for 3 min. Similarly, the other five groups were administered using the same procedure. After different treatments, the body weight of mice was recorded and the wound sizes of mice in all groups were measured perpendicularly ($d_1 \leq d_2$) using the digital caliper. The wound area (S) was calculated according to the following equation: $S = \pi d_1 d_2/4$. (Huo et al., 2019) At 5 days, the skin samples on mice were dissected for histopathological H&E staining and Masson's trichrome staining analysis. Additionally, any mouse of each group was dissected and the main organs (heart, liver, spleen, lung, and kidney) were collected for hematoxylin and eosin (H&E) staining analysis of biocompatibility. (Wang et al., 2018)

***In vivo* toxicity, biodistribution, and metabolism:** The *in vivo* toxicity of ND nanozymes was preliminarily evaluated by serum biochemistry and a complete blood panel test. In brief, ND nanozymes (10 mg/kg, 100 μL) were intravenously injected into three groups (five mice per group) of healthy Balb/c mice. At the 1st, 7th, and 15th day after injection, all five mice in each group were sacrificed to collect blood samples (~1.0 mL) for serum biochemistry and complete blood panel test. Another five healthy Balb/c mice treated only with PBS were used as control. The major organs (heart, liver, spleen, lung, and kidney) of those mice were dissected and divided into two halves for histological examination and biodistribution measurement, respectively. For histological examination, any mouse of each group was dissected and the main organs were collected for hematoxylin and eosin (H&E) staining. For biodistribution measurement, the major organs from the mice were solubilized by aqua regia for inductively coupled plasma-optical emission spectroscopy (ICP-OES) measurement to determine the Ni levels in these different organs. Additionally, to study the metabolic pathway, BALB/c mice intravenously injected with the ND nanozymes (10 mg/kg, 100 μL) were placed in the metabolism cages to collect their urine and fences at different time points, then solubilized by aqua regia for ICP-OES measurement to determine the Ni contents.

Supporting Figures

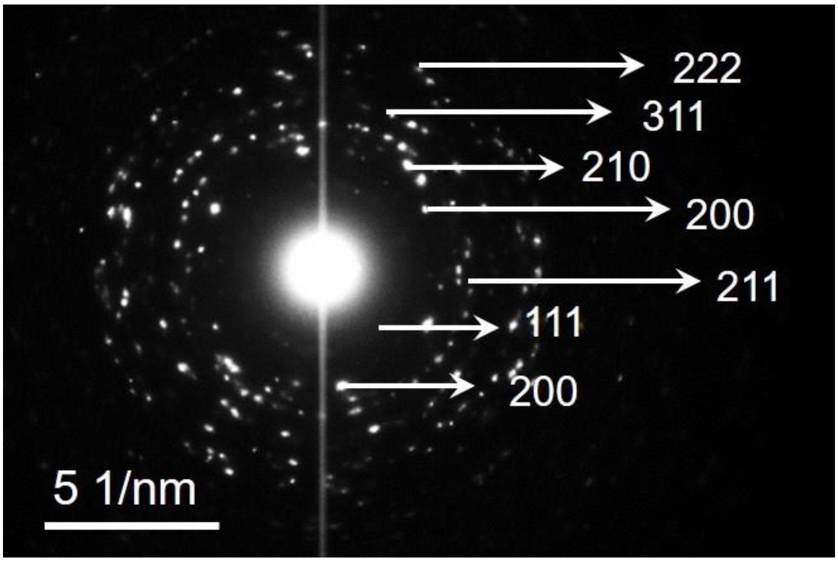

Figure S1. SAED image of the ND nanozymes. Related to Figure 1.

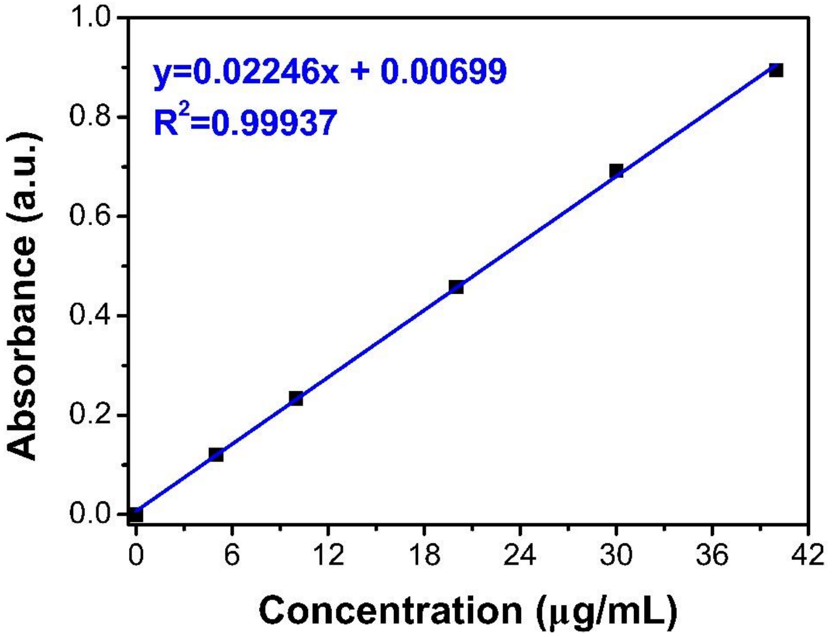

Figure S2. The mass extinction coefficient of the ND nanozymes. Related to Figure 2.

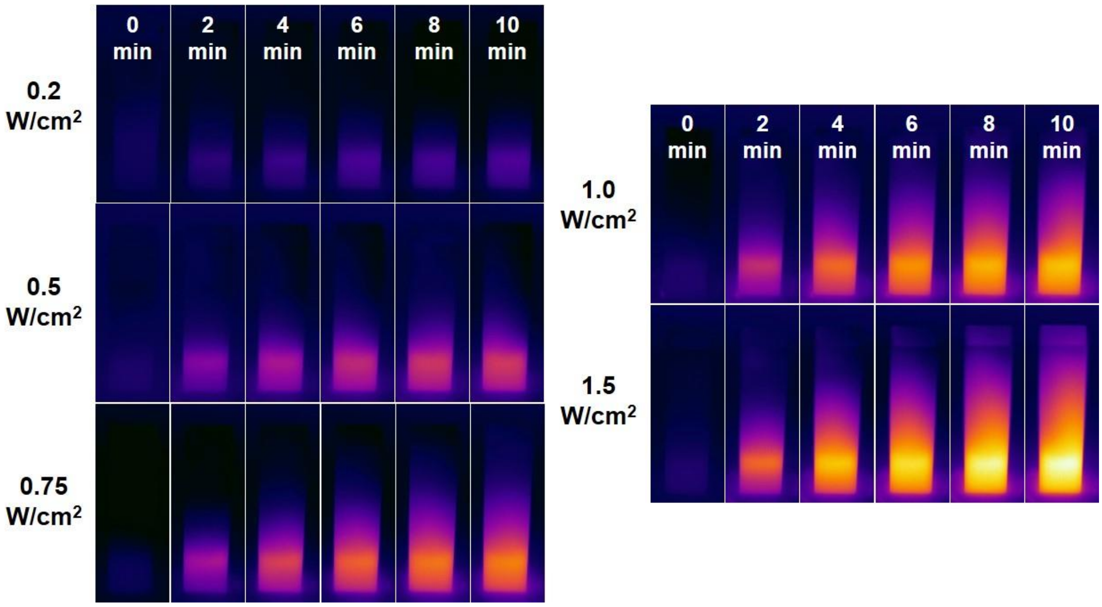

Figure S3. Thermal images of ND nanozymes solution under 808 nm laser irradiation at different power densities (0.2, 0.5, 0.75, 1.0, and 1.5 W/cm^2) for 10 min. Related to Figure 2.

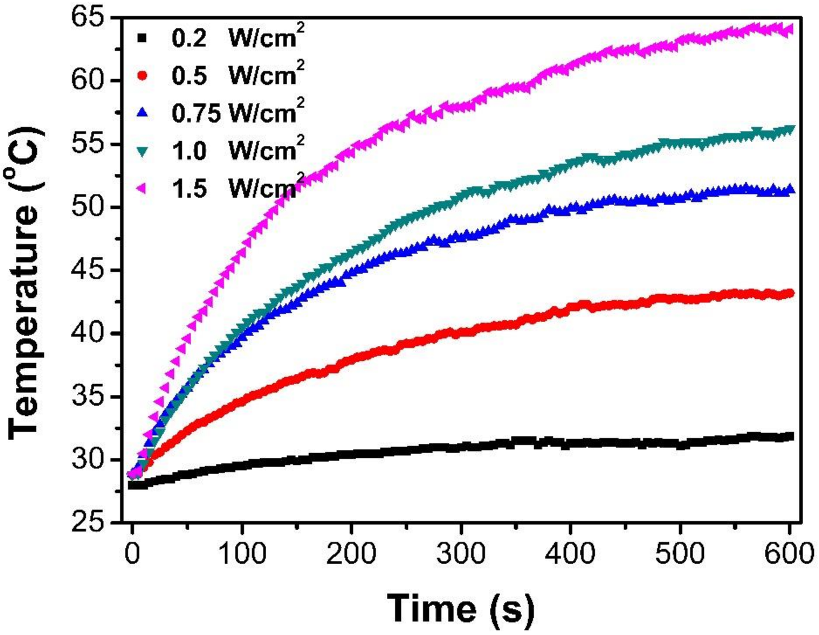

Figure S4. Temperature change curves of the ND nanozymes solution under 808 nm laser irradiation at different power densities (0.2, 0.5, 0.75, 1.0, and 1.5 W/cm^2) for 10 min. Related to Figure 2.

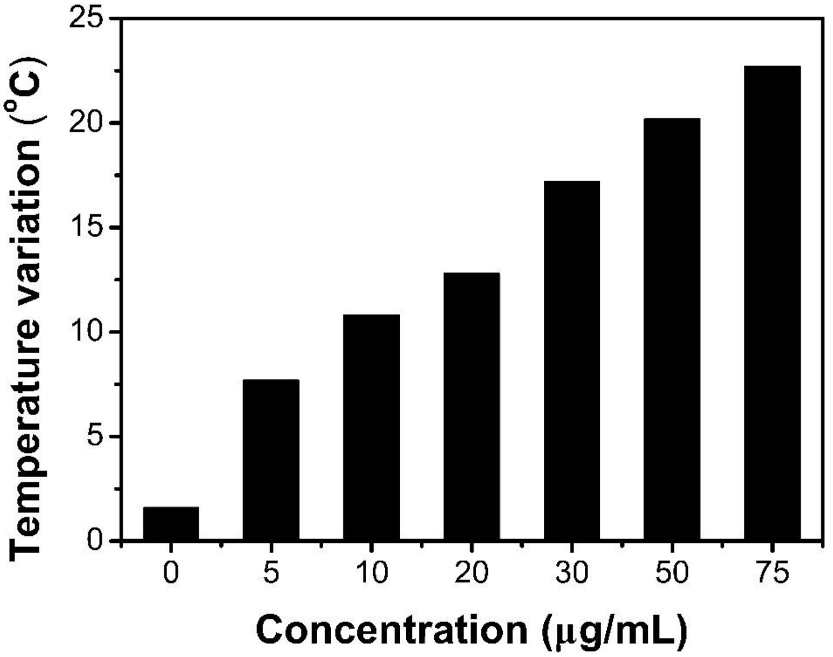

Figure S5. The plot of temperature variation over 10 min with different concentration of the ND nanozymes. Related to Figure 2.

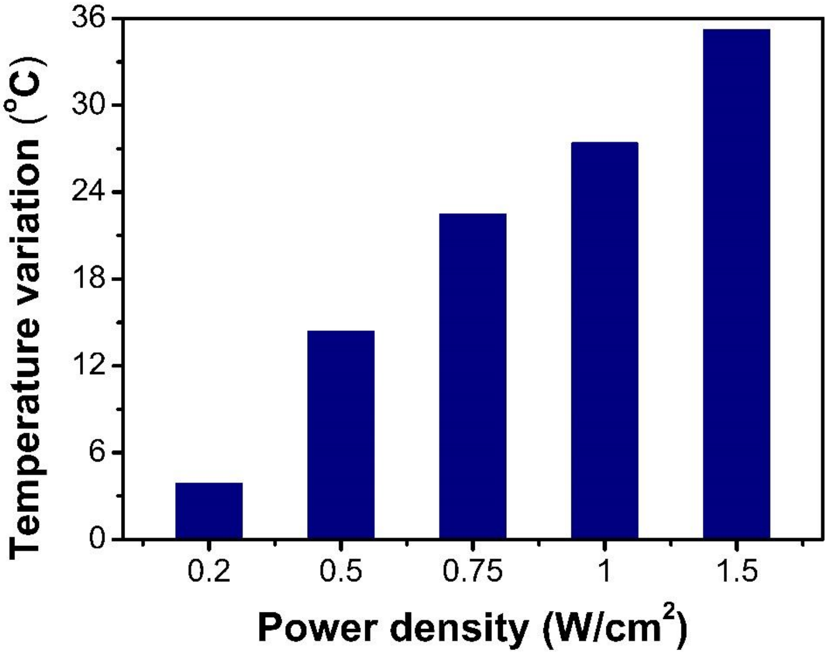

Figure S6. The plot of temperature variation over 10 min of ND nanozymes and with different power densities of 808 nm laser. Related to Figure 2.

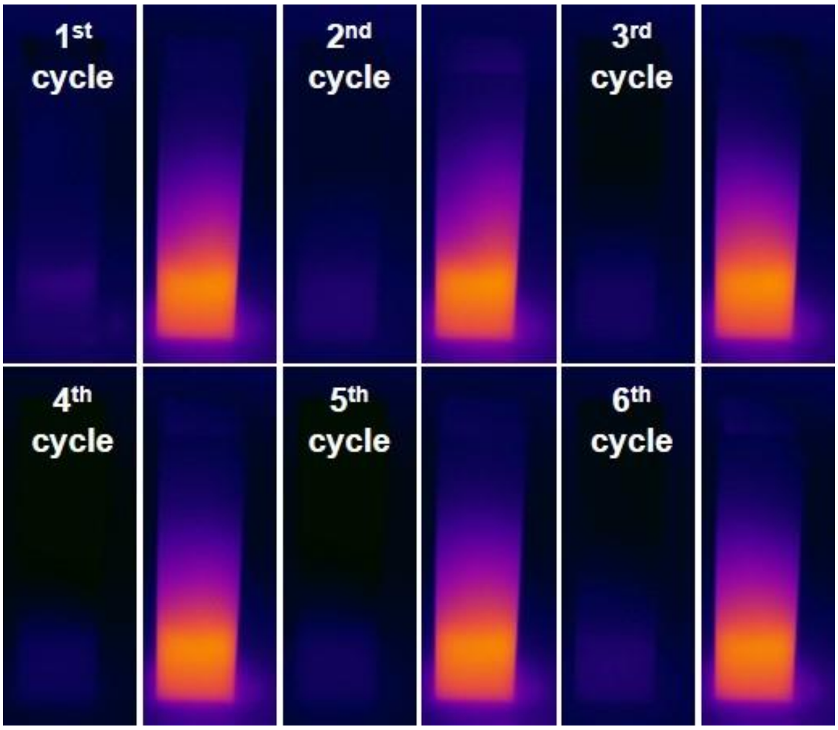

Figure S7. Starting temperature and maximum temperature of six cycles of laser irradiation. Related to Figure 2.

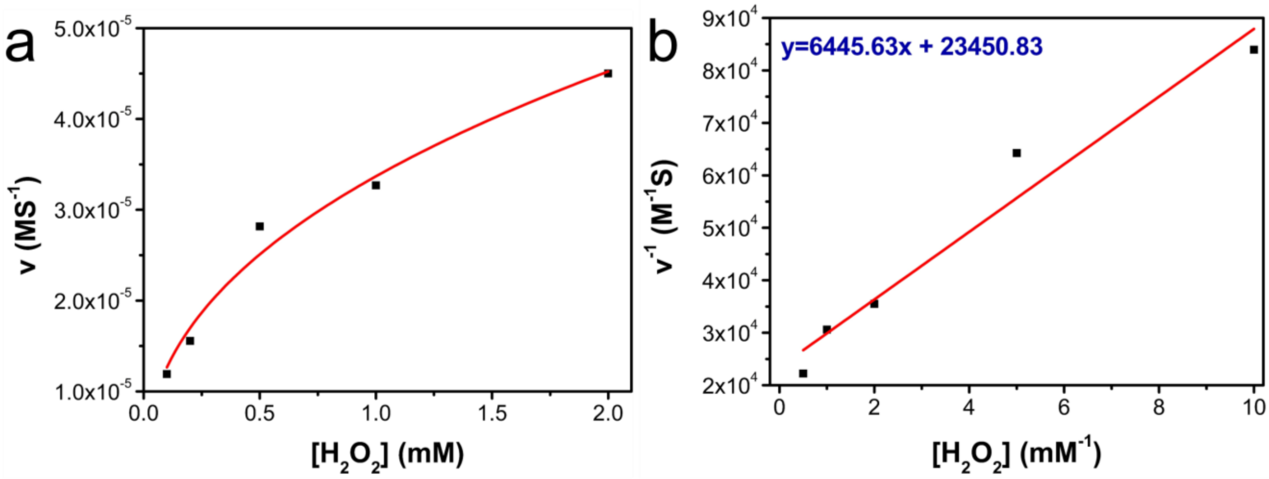

Figure S8. (a)The Michaelis-Menten fitting curves of initial hydroxyl radical generation velocities versus H_2O_2 concentration; (b) the Lineweaver-Burke fitting (double reciprocal) of Michaelis-Menten fitting curve. Related to Figure 3.

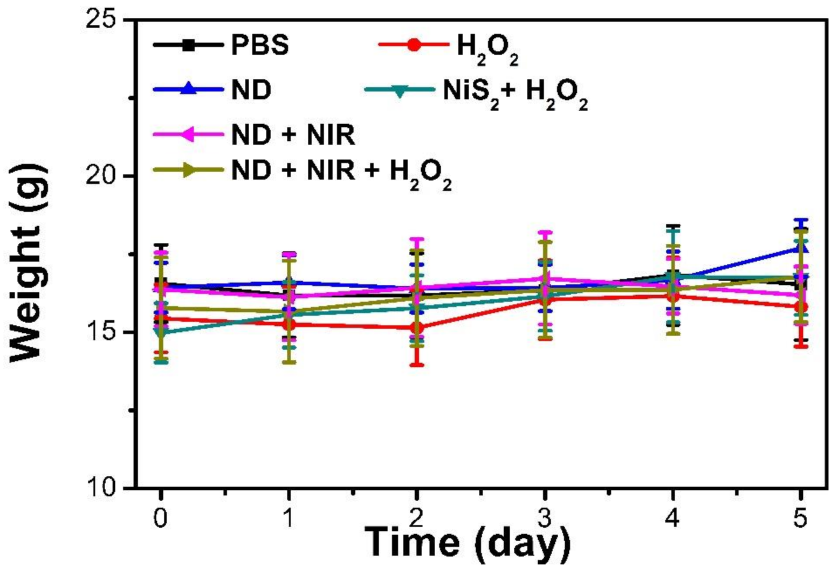

Figure S9. The body weights change during various treatments. Related to Figure 7.

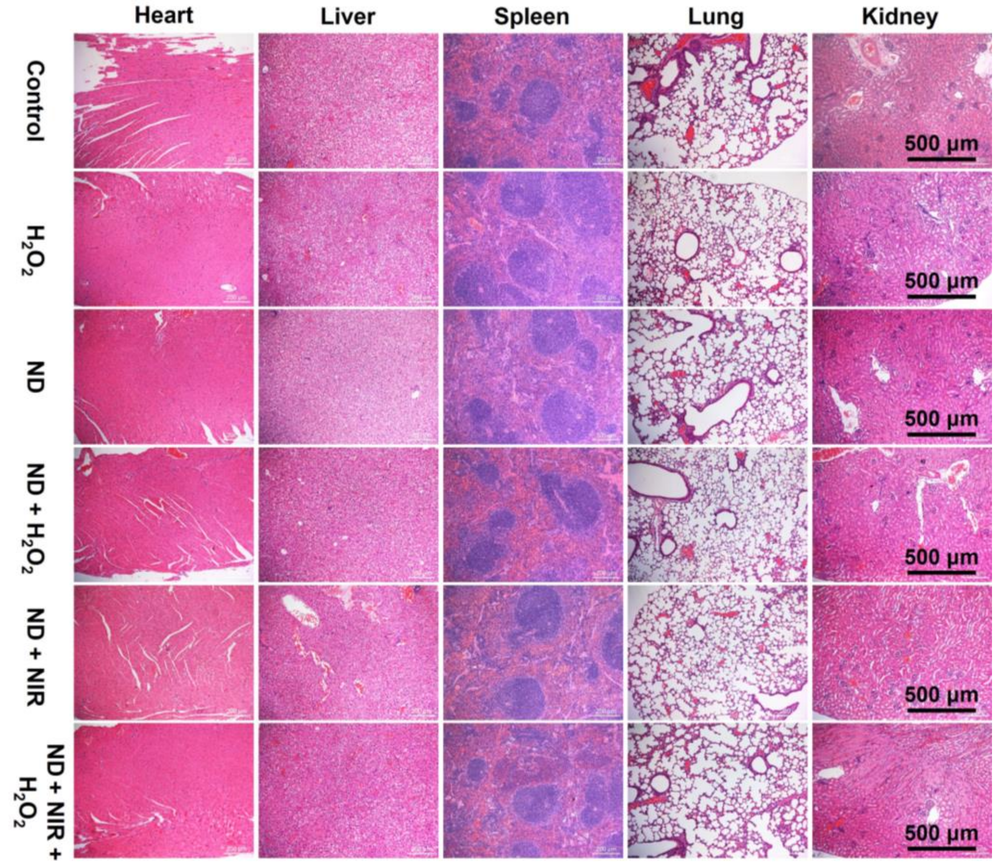

Figure S10. Toxicity evaluation of the ND nanozymes after antibacterial treatment *in vivo.* The H&E staining of the major organs (heart, liver, spleen, lung, and kidney) from mice after different treatments. Related to Figure 7.

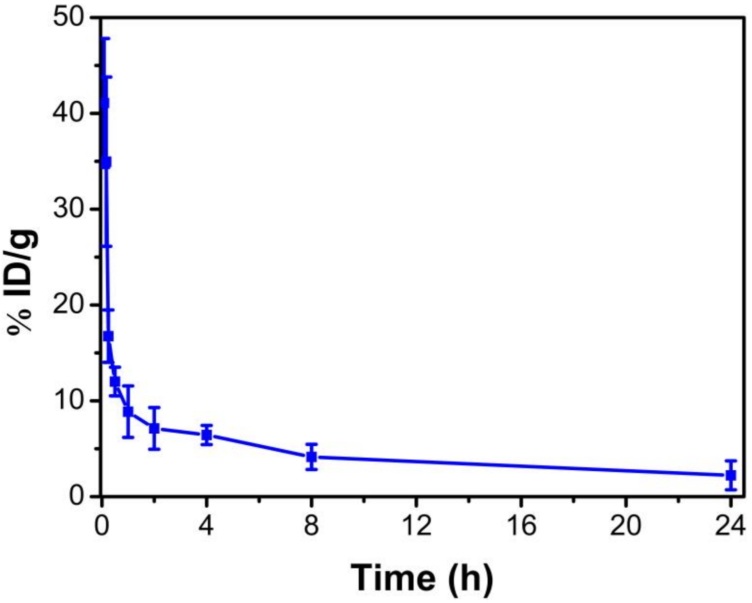

Figure S11. Blood circulation of ND nanozymes post i.v. injection. Related to Figure 8.

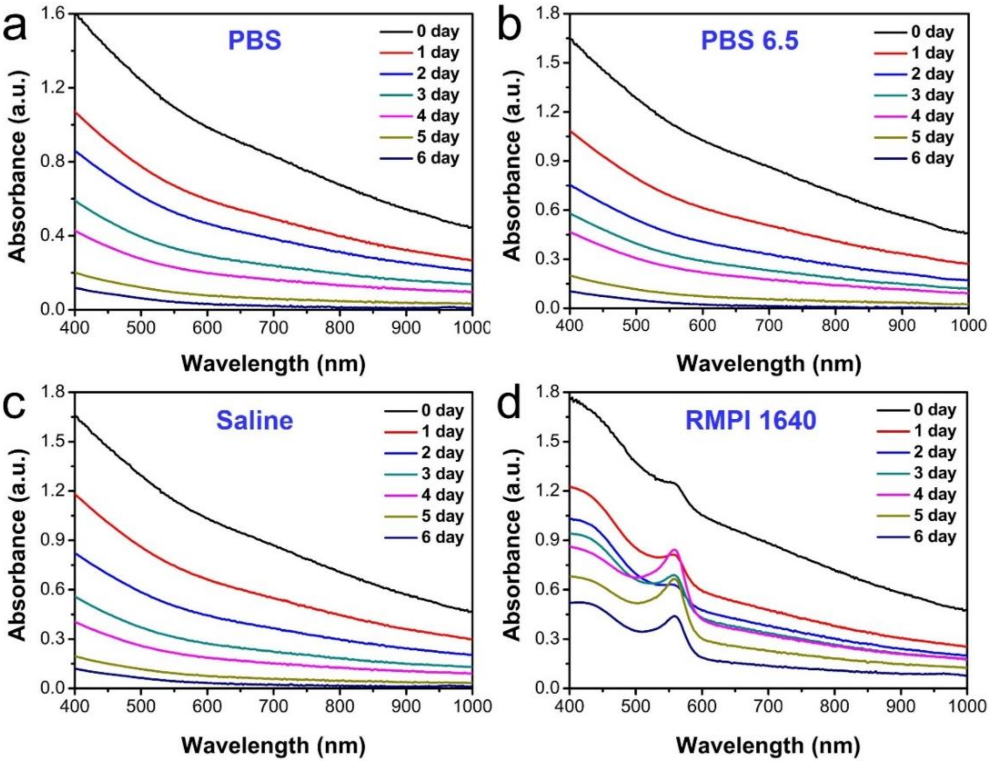

Figure S12. UV-vis-NIR absorption spectra of the ND nanozymes in (a) PBS, (b) PBS 6.5, (c) normal saline, (d) RMPI 1640 over time. Related to Figure 8.

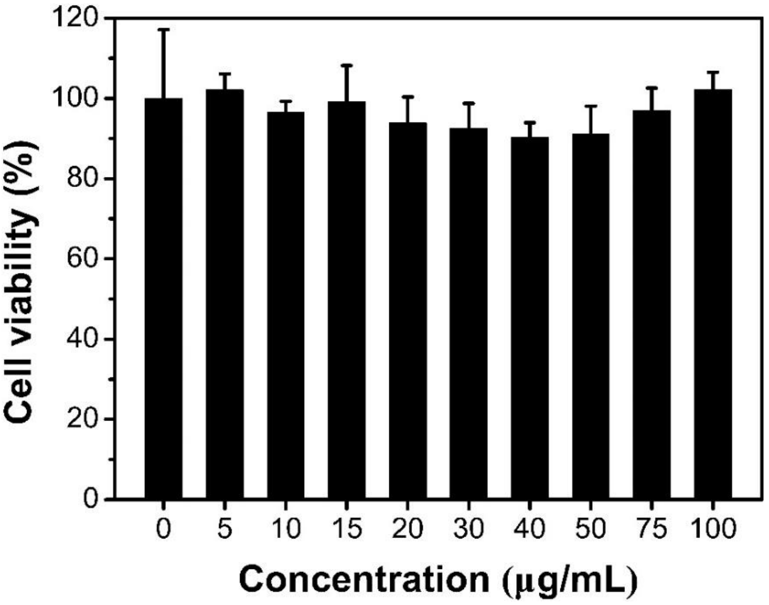

Figure S13. The relative cell viability of HUEVC after incubation with ND nanozymes. Related to Figure 9.

Table S1 Comparison of photothermal conversion efficiency. Related to Figure 2.

**Materials**	**Photothermal conversion efficiency**	**Ref**
Cu_2Te	33.1%	(Wang et al., 2017)
Cu_9S_5	25.7%	(Tian et al., 2011)
Prussian blue	32.2%	(Feng et al., 2019)
WS_2	32.83%	(Yong et al., 2014)
Graphene	25.9%	(Neelgund and Oki, 2016)
Black phosphorus	28.4%	(Sun et al., 2015)
Au@TiO_2	42.05%	(Gao et al., 2019)
Ti_3C_2	28.3%	(Tang et al., 2019)
V_2C	45.5%	(Cao et al., 2019)
Pt	52.5%	(Wang et al., 2019a)
Hollow MoS_2	34.46%	(Tan et al., 2016)
**ND nanozymes**	**43.8 %**	**This work**

References:

Cao, Y., Wu, T., Zhang, K., Meng, X., Dai, W., Wang, D., Dong, H., and Zhang, X. (2019). Engineered Exosome-Mediated Near-Infrared-II Region V_2C Quantum Dot Delivery for Nucleus-Target Low-Temperature Photothermal Therapy. ACS Nano *13*, 1499-1510.

Feng, T., Wan, J., Li, P., Ran, H., Chen, H., Wang, Z., and Zhang, L. (2019). A novel NIR-controlled NO release of sodium nitroprusside-doped Prussian blue nanoparticle for synergistic tumor treatment. Biomaterials *214*, 119213.

Gao, F., He, G., Yin, H., Chen, J., Liu, Y., Lan, C., Zhang, S., and Yang, B. (2019). Titania-coated 2D gold nanoplates as nanoagents for synergistic photothermal/sonodynamic therapy in the second near-infrared window. Nanoscale *11*, 2374-2384.

Guan, Q., Fu, D., Li, Y., Kong, X., Wei, Z., Li, W., Zhang, S., and Dong, Y. (2019). BODIPY-decorated nanoscale covalent organic frameworks for photodynamic therapy. iScience *14*, 180-198.

He, G., Ma, Y., Zhou, H., Sun, S., Wang, X., Qian, H., Xu, Y., Miao, Z., and Zha, Z. (2019). Mesoporous NiS_2 nanospheres as a hydrophobic anticancer drug delivery vehicle for synergistic photothermal-chemotherapy. J. Mater. Chem. B *7*, 143-149.

Huo, M., Wang, L., Zhang, H., Zhang, L., Chen, Y., and Shi, J. (2019). Construction of single-iron-atom nanocatalysts for highly efficient catalytic antibiotics. Small *15*, 1901834.

Liu, Y., Guo, Z., Li, F., Xiao, Y., Zhang, Y., Bu, T., Jia, P., Zhe, T., and Wang, L. (2019). Multifunctional magnetic copper ferrite nanoparticles as Fenton-like reaction and near-infrared photothermal agents for synergetic antibacterial therapy. ACS Appl. Mater. Interfaces *11*, 31649-31660.

Miao, Z., Fan, L., Xie, X., Ma, Y., Xue, J., He, T., and Zha, Z. (2019). Liquid exfoliation of atomically thin antimony selenide as an efficient two-dimensional antibacterial nanoagent. ACS Appl. Mater. Interfaces *11*, 26664-26673.

Neelgund, G.M., and Oki, A.R. (2016). Influence of carbon nanotubes and graphene nanosheets on photothermal effect of hydroxyapatite. J. Colloid Interface Sci. *484*, 135-145.

Sun, Z., Xie, H., Tang, S., Yu, X.-F., Guo, Z., Shao, J., Zhang, H., Huang, H., Wang, H., and Chu, P.K. (2015). Ultrasmall Black Phosphorus Quantum Dots: Synthesis and Use as Photothermal Agents. Angew. Chem. Inter. Ed. *54*, 11526-11530.

Tan, L., Wang, S., Xu, K., Liu, T., Liang, P., Niu, M., Fu, C., Shao, H., Yu, J., Ma, T., *et al.* (2016).

Layered MoS_2 Hollow Spheres for Highly-Efficient Photothermal Therapy of Rabbit Liver Orthotopic Transplantation Tumors. Small *12*, 2046-2055.

Tang, W., Dong, Z., Zhang, R., Yi, X., Yang, K., Jin, M., Yuan, C., Xiao, Z., Liu, Z., and Cheng, L. (2019). Multifunctional Two-Dimensional Core–Shell MXene@Gold Nanocomposites for Enhanced Photo–Radio Combined Therapy in the Second Biological Window. ACS Nano *13*, 284-294.

Tian, Q., Jiang, F., Zou, R., Liu, Q., Chen, Z., Zhu, M., Yang, S., Wang, J., Wang, J., and Hu, J. (2011). Hydrophilic Cu_9S_5 Nanocrystals: A Photothermal Agent with a 25.7% Heat Conversion Efficiency for Photothermal Ablation of Cancer Cells in Vivo. ACS Nano *5*, 9761-9771.

Wan, S.-S., Cheng, Q., Zeng, X., and Zhang, X.-Z. (2019). A Mn(III)-sealed metal-organic framework nanosystem for redox-unlocked tumor theranostics. ACS Nano *13*, 6561-6571.

Wang, D., Shi, R., Zhou, J., Shi, S., Wu, H., Xu, P., Wang, H., Xia, G., Barnhart, T.E., and Cai, W. (2018). Photo-enhanced singlet oxygen generation of Prussian blue-based nanocatalyst for augmented photodynamic therapy. iScience *9*, 14-26.

Wang, Q., Wang, H., Yang, Y., Jin, L., Liu, Y., Wang, Y., Yan, X., Xu, J., Gao, R., Lei, P.*, et al.* (2019a). Plasmonic Pt Superstructures with Boosted Near-Infrared Absorption and Photothermal Conversion Efficiency in the Second Biowindow for Cancer Therapy. Adv. Mater. *31*, 1904836.

Wang, X., Ma, Y., Chen, H., Wu, X., Qian, H., Yang, X., and Zha, Z. (2017). Novel doxorubicin loaded PEGylated cuprous telluride nanocrystals for combined photothermal-chemo cancer treatment. Colloids Surf., B *152*, 449-458.

Wang, X., Zhong, X., Lei, H., Geng, Y., Zhao, Q., Gong, F., Yang, Z., Dong, Z., Liu, Z., and Cheng, L. (2019b). Hollow Cu_2Se nanozymes for tumor photothermal-catalytic therapy. Chem. Mater. *31*, 6174-6186.

Wang, X., Zhong, X., Zha, Z., He, G., Miao, Z., Lei, H., Luo, Q., Zhang, R., Liu, Z., and Cheng, L. (2020). Biodegradable CoS_2 nanoclusters for photothermal-enhanced chemodynamic therapy. Appl. Mater. Today *18*, 100464.

Yong, Y., Zhou, L., Gu, Z., Yan, L., Tian, G., Zheng, X., Liu, X., Zhang, X., Shi, J., Cong, W.*, et al.* (2014). WS_2 nanosheet as a new photosensitizer carrier for combined photodynamic and photothermal therapy of cancer cells. Nanoscale *6*, 10394-10403.
